# A compartmentalized SCN model explains temperature resistance through interregional phase differences

**DOI:** 10.1371/journal.pcbi.1014831

**Published:** 2026-09-29

**Authors:** Feng Yu, Ling Yang, Jie Yan

**Affiliations:** School of Mathematical Sciences, Soochow University, Suzhou, China; National Institutes of Health, UNITED STATES OF AMERICA

## Abstract

Temperature cycles entrain circadian rhythms in many peripheral tissues, yet the master circadian pacemaker (suprachiasmatic nucleus) in mammals remains largely insensitive to the same input. Despite their similar molecular circuitry, the mechanisms underlying the distinct temperature responses of central and peripheral clocks remain unclear. To address this, we developed a compartmentalized model of the SCN circadian clock that integrates the heat shock protein (HSP) pathway, simulating the response of the SCN network to temperature variations. Using this model, we successfully recapitulated key experimental observations, including the ability of heat stimuli to entrain peripheral clocks to an inverted phase while the SCN maintains its resistance. Further analysis reveals that intrinsic phase heterogeneity among SCN subregions underlies this entrainment resistance. Specifically, the phase difference between the dorsal and ventral SCN leads to divergent phase responses upon simultaneous stimulation, resulting in an attenuated net shift of the overall SCN phase. Moreover, the mode of thermal input, in which the dorsal and ventral regions are stimulated concurrently, may further enhance the SCN’s robustness to temperature entrainment. These results suggest that the SCN’s resistance to temperature entrainment may differ mechanistically from photic entrainment, and highlight the potential importance of its spatial structure in maintaining circadian stability.

## Author summary

Daily temperature cycles help synchronize biological clocks with the external environment. In many peripheral tissues, these thermal rhythms can strongly shift clock phase. In contrast, the suprachiasmatic nucleus (SCN), the master circadian pacemaker in mammals, remains resistant to temperature entrainment. Why the SCN and peripheral clocks respond so differently to the same thermal signals has remained unclear. We developed a mathematical model of the SCN that combines temperature-responsive signaling with the spatial organization of the SCN network. Our simulations show that the SCN’s resistance to temperature entrainment arises from an intrinsic phase difference between dorsal and ventral regions. When the same thermal input reaches both subregions, their local phase responses diverge and partially cancel, thereby buffering the phase of the SCN as a whole. Therefore, this built-in spatial heterogeneity helps protect the SCN from temperature perturbation. These results provide a mechanistic explanation for the thermal robustness of the SCN and highlight how spatial organization within a biological network can stabilize physiological rhythms. More broadly, one reason the SCN naturally maintains a distributed phase structure may be that such heterogeneity enhances robustness to temperature perturbation.

## Introduction

The circadian clock is a fundamental timekeeping mechanism present in a wide range of organisms, where it plays a crucial role in orchestrating the orderly timing of various biological activities [1–3]. In mammals, circadian organization is hierarchical. The principal pacemaker is located in the suprachiasmatic nucleus (SCN) of the anterior hypothalamus, while peripheral clocks are present in nearly all tissues and organs, where they regulate local rhythms of gene expression and physiological activity. These peripheral oscillators can be modulated by external cues such as feeding schedules and temperature cycles [4,5]. Signals from the SCN coordinate peripheral clocks through neurohumoral pathways, thereby maintaining circadian alignment across the organism under changing environmental conditions [6].

The SCN contains approximately 20,000 densely packed neurons arranged bilaterally above the optic chiasm on either side of the third ventricle [7]. It is commonly divided into ventrolateral and dorsomedial subregions, also referred to as the core and shell, respectively. These subregions are connected through an organized network of intercellular coupling that supports coherent circadian oscillations across the SCN [8]. Gamma-aminobutyric acid (GABA) is broadly expressed throughout the SCN and contributes to intercellular communication, whereas region-specific neuropeptides define distinct functional identities within the network [9]. In particular, the ventrolateral SCN is enriched in vasoactive intestinal peptide (VIP), while the dorsomedial SCN is characterized by arginine vasopressin (AVP) expression [10]. VIP released from ventral SCN neurons can regulate clock gene expression in dorsal SCN neurons and thereby serves as an important mediator of interregional coupling [11]. Experimental studies have shown that SCN subregions can maintain distinct circadian phases, with phase differences approaching 8–10 hours [12,13]. This internal phase structure is thought to contribute to the temporal organization of SCN output and downstream physiological rhythms.

At the cellular level, circadian rhythms are generated by a conserved transcriptional and translational feedback loop. In this core circuit, the BMAL1:CLOCK complex activates transcription of *Period* (*Per1* and *Per2*) and *Cryptochrome* (*Cry1* and *Cry2*) genes, while the resulting PER and CRY proteins accumulate, form complexes, and feed back to repress BMAL1:CLOCK activity. This delayed negative feedback generates approximately 24-hour oscillations in both the SCN and peripheral tissues. At the same time, circadian oscillators are modulated by environmental inputs. Among these, temperature fluctuations can reset circadian rhythms, particularly in peripheral tissues [14–18]. Cellular responses to temperature change are mediated in part by heat shock proteins (HSPs) and the transcription factor heat shock factor (HSF1). Notably, HSF1 can stimulate *Per2* expression and physically interact with the BMAL1:CLOCK complex, thereby providing a direct link between temperature-responsive signaling and the core circadian oscillator [19,20].

Despite the broad conservation of core clock machinery and temperature-responsive signaling pathways, experimental studies have revealed a marked difference between the SCN and peripheral tissues in their responses to thermal entrainment. Takahashi and colleagues showed that circadian clocks in peripheral tissues such as pituitary and lung can be reset by inverted temperature cycles, leading to phase reversal, whereas the phase of the intact SCN remains largely unchanged under the same conditions. Remarkably, when the ventrolateral and dorsomedial regions of the SCN are physically separated, the SCN becomes susceptible to temperature-induced phase shifts [21]. These findings suggest that resistance to thermal entrainment in the intact SCN is not simply a property of individual cellular oscillators, but rather an emergent property of the coupled network. However, how spatial organization, interregional coupling, and internal phase structure combine to produce this robustness remains unclear. Mathematical modeling provides a powerful tool for understanding the dynamical mechanisms that govern circadian rhythms. A large body of circadian models has been developed to describe the core transcriptional and translational feedback loop, its oscillatory properties, and its responses to external entrainment cues. At the molecular level, such models have clarified how feedback architecture, transcriptional regulation, and time delays generate robust circadian oscillations [22–27]. At the network level, modeling studies of the SCN have generally focused on how neuronal heterogeneity and coupling contribute to synchrony, phase coherence, and the robustness of population rhythms [28,29]. For example, previous work has shown that the synchronized period of the SCN emerges from the interaction between coupling and intracellular transcriptional feedback regulation [29]. Other studies have considered the effects of environmental cues, including light [30], feeding schedules [31], and temperature [32], on circadian entrainment in single oscillators or peripheral tissues. However, comparatively few models have addressed how spatial organization within the SCN shapes network responses to thermal input. The network-level basis of SCN resistance to temperature entrainment remains insufficiently understood.

To address this question, we developed a compartmentalized mathematical model that integrates temperature-responsive signaling with regional structure in the SCN. The model represents dorsal and ventral SCN subregions as coupled compartments occupying different circadian phases, allowing us to examine how internal phase offsets shape tissue-level responses to thermal input. Our results support a mechanism in which spatial phase heterogeneity within the SCN limits net phase resetting and thereby stabilizes circadian timing against temperature perturbation.

## Materials and methods

Our approach integrates a temperature signaling cascade with a circadian clock gene network in a coupled ordinary differential equation model, allowing us to examine how temperature rhythms influence circadian entrainment. We considered two configurations. The first is a model without spatial heterogeneity, used to examine temperature entrainment in the absence of regional structure and to represent peripheral circadian clocks. The second is a compartmentalized SCN model, in which the dorsal and ventral regions are represented as distinct but coupled compartments under temperature regulation. This compartmentalized formulation was motivated by experimental observations showing that the intact SCN remains resistant to inverted temperature cycles while resistance is lost when the dorsomedial and ventrolateral regions are physically separated in coronal SCN slices [21].

### Temperature-regulated circadian clock model without spatial heterogeneity

We first considered a temperature-regulated circadian clock model without spatial heterogeneity. This formulation couples the temperature signaling cascade to the core circadian clock gene network and provides the common modeling framework used in both the homogeneous and compartmentalized settings. In the absence of regional subdivision, the model can also serve as an effective description of peripheral circadian clocks. The corresponding dynamics are described by a system of coupled ordinary differential equations for temperature-responsive signaling and clock gene regulation.

In the framework, we first specify the temperature-responsive signaling pathway that drives thermal input to the circadian clock (Fig 1A). Cellular responses to sudden temperature fluctuations can be mediated by HSPs. In unstressed cells, HSPs bind HSF1, forming an inactive HSF1:HSP complex. Upon exposure to heat stress, denatured proteins accumulate, prompting HSPs to dissociate from HSF1 and bind these misfolded proteins instead. The released HSF1 then trimerizes into its active form and translocates to the nucleus, where it binds to heat shock elements (HSEs) and activates transcription of heat shock genes [33]. As levels of HSPs rise, they in turn facilitate the conversion of active HSF1 trimers back to inactive monomers, decreasing transcriptional activity and returning HSF1 to its basal distribution. This negative feedback loop allows HSF1, under the regulation of HSPs, to sense and respond to cellular stress, thereby maintaining proteostasis. Meanwhile, once activated HSF1 trimers are formed during this process, they can directly promote the transcription of *Per2* mRNA by binding to HSEs within the *Per2* gene. Also, HSF1 can interact with the BMAL1:CLOCK complex, thereby attenuating *E-box*-mediated transcription [20]. Together, these pathways enable the circadian clock to sense and respond to temperature signals. To describe the temperature-dependent heat shock response, we adopted a mathematical model developed by Szymańska and Zylicz for heat shock protein synthesis under varying thermal conditions [33]. We then coupled this module to an established circadian clock model [34] by modifying the *Per2* mRNA equation. In particular, we added an HSF1-trimer-dependent activation term to represent HSF1-mediated induction of *Per2* transcription. Therefore, the dynamics of *Per2* mRNA included the basal transcription, BMAL1:CLOCK-dependent transcriptional activation, repression by PER:CRY complexes, HSF1-trimer-mediated induction, and first-order degradation. To simplify notation, all activating and inhibitory regulatory terms were written using common Hill functions g(·) and f(·), respectively. The resulting equation is


$$\begin{array}{cc} \frac{d\left[m_{Per2}\right]}{dt}= & \left(V_{0,P2}+V_{1,P2}g_{\alpha 1,P2}(\left[BC\right])\right)f_{I1,P2}(\left[P1C1\right])f_{I2,P2}(\left[P2C1\right])f_{I3,P2}(\left[P1C2\right]),\\ & f_{I4,P2}(\left[P2C2\right])-k_{m,P2}\cdot \left[m_{Per2}\right]+V_{2,HSF1_{3}}g_{3,HSF1_{3}}(\left[{HSF1}_{3}\right]), \end{array}$$

**Fig 1:**
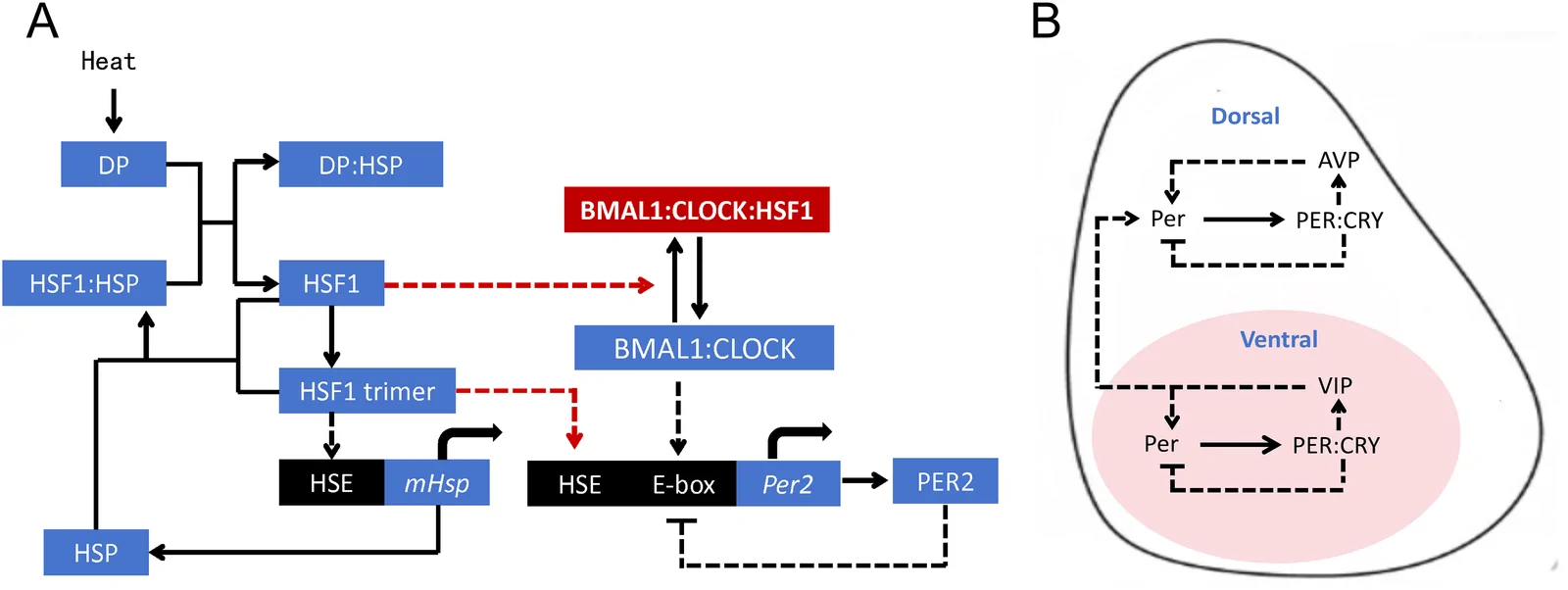
Schematic illustration of the two model architectures. **A**: Homogeneous temperature-regulated clock model. A single compartment model integrates the heat shock signaling cascade with the core circadian clock network, representing a spatially uniform peripheral oscillator entrained by thermal input. **B**: Compartmentalized SCN clock model. The SCN consists of ventral and dorsal compartments, each with a local clock network. The two regions are connected by peptide signaling: ventral VIP regulates both regions, and dorsal AVP acts locally. These signals modulate *Per* expression and interregional phase relationships, allowing analysis of how spatial organization influences SCN thermal responses.

where $g_{i,j}(\left[X\right])=\frac{{\left[X\right]}^{n_{i,j}}}{K_{i,j}^{n_{ij}}+{\left[X\right]}^{n_{i,j}}},\;f_{i,j}(\left[X\right])=\frac{K_{i,j}^{n_{ij}}}{K_{i,j}^{n_{ij}}+{\left[X\right]}^{n_{i,j}}}$.

Here, [BC] denotes the BMAL1:CLOCK complex, $\left[{HSF1}_{3}\right]$ denotes HSF1 trimers, and [P1C1], [P1C2], [P2C1], and [P2C2] denote the inhibitory PER:CRY complexes PER1:CRY1, PER1:CRY2, PER2:CRY1, and PER2:CRY2, respectively.

HSF1 can also regulate the circadian clock through interaction with the BMAL1:CLOCK complex [20]. To represent this process, we introduced a new variable, [HBC], denoting the complex formed between HSF1 and BMAL1:CLOCK.


$$\begin{array}{c} \frac{d\left[HBC\right]}{dt}=\alpha_{2}\;\left[BC\right]\cdot \left[HSF1\right]-\delta_{2}\;\left[HBC\right]. \end{array}$$

The HSF1 and BMAL1:CLOCK equations were modified accordingly to include the formation and dissociation of the HBC complex. Specifically, the terms $-\alpha_{2}\left[BC\right]\cdot \left[HSF1\right]$ and $+\delta_{2}\left[HBC\right]$ represent the reversible association and dissociation of the BMAL1:CLOCK:HSF1 complex. All other terms in these equations describe synthesis, degradation, chaperone interactions, and oligomerization dynamics inherited from the original model [33].


$$\begin{array}{cc} \frac{d\left[BC\right]}{dt} & =\alpha_{1}\;\left[CLK\right]\cdot \left[BMAL1\right]-\delta_{1}\;\left[BC\right]-\alpha_{2}\;\left[BC\right]\cdot \left[HSF1\right]+\delta_{2}\;\left[HBC\right],\\ {}[6pt]\frac{d\left[HSF1\right]}{dt} & =-k_{1}\;\left[hsp\right]\cdot \left[HSF1\right]+\ell_{1}\;\left[hspHSF1\right]-3k_{3}\;{\left[HSF1\right]}^{3}+2\ell_{3}\;\left[hsp\right]\cdot \left[{HSF1}_{3}\right]\\ & \;+k_{6}\;\left[hspHSF1\right]\cdot \left[S\right]-\ell_{6}\;\left[hspS\right]\cdot \left[HSF1\right]-\alpha_{2}\;\left[BC\right]\cdot \left[HSF1\right]+\delta_{2}\;\left[HBC\right]. \end{array}$$

The effect of temperature was incorporated into the heat shock signaling module using a temperature-dependent function that has been approximated by Peper et al. [35]. Here, T denotes the external temperature. This function was used to modulate HSF1 activation in response to temperature changes.


$$\begin{array}{c} I=0.03\cdot \left(1-0.4\;e^{-(T-37)}\right)\cdot 1.4^{\;T-37}. \end{array}$$

In the present model, the newly introduced HSF1–clock coupling parameters were treated as effective phenomenological terms, since these interaction strengths are difficult to estimate directly from currently available experimental data. In this formulation, heat stimulation regulates *Per2* expression through two competing routes: direct activation by HSF1 trimers and indirect attenuation of BMAL1:CLOCK-dependent transcription through formation of the HSF1:BMAL1:CLOCK complex. Because experimental studies show a rapid increase in *Per2* expression following heat stimulation, the reference parameter set was specified so that the direct HSF1-trimer-mediated activation dominates the indirect inhibitory effect of BMAL1:CLOCK sequestration. The experimentally observed acute induction of *Per2* served as the main biological constraint in selecting these heat-response coupling parameters. To further assess the robustness of this parameter selection, all newly introduced parameters were evaluated jointly, as described below and in Section B of S1 Text.

Except for the additional coupling terms described above, the remaining equations follow the original circadian clock model [34] and the HSP:HSF1 interaction framework [33]. The complete equations and parameter values are given in Section A of S1 Text. Representative autonomous oscillations of the peripheral clock model at a constant 37°C are shown in S1 Fig, including the mRNA time courses of the core clock genes *Bmal1*, *Per1*, *Per2*, *Cry1*, *Cry2*, and *Rev-erb*α.

### A compartmentalized model of the SCN’s circadian clock with temperature regulation

We next extended the framework described above to a compartmentalized model of the SCN, in which the ventral and dorsal regions are represented as distinct but coupled compartments. Each compartment contains the same core circadian clock network and a local temperature-responsive signaling pathway, allowing thermal input to regulate the ventral and dorsal clocks separately while preserving explicit regional phase differences. Interregional communication was introduced through peptide-mediated signaling. Specifically, the ventral SCN predominantly expresses VIP, whereas the dorsal SCN is characterized by AVP expression [10]. In the model, VIP release is regulated by PER:CRY complexes in the ventral compartment, while AVP release is regulated by PER:CRY complexes in the dorsal compartment [36,37]. AVP promotes *Per1* and *Per2* transcription only in the dorsal compartment, whereas VIP promotes *Per1* and *Per2* transcription in both ventral and dorsal compartments through CREB-mediated activation at CRE sites [10,38]. Thus, the coupling is asymmetric: VIP, produced in the ventral compartment, acts on both ventral and dorsal clocks, whereas AVP, produced in the dorsal compartment, acts only within the dorsal clock (Fig 1B). To represent these interactions, we introduced two variables corresponding to VIP and AVP concentrations. The dynamics of VIP and AVP are given by the following equations.


$$\begin{array}{cc} \frac{d\left[VIP\right]}{dt}= & \;k_{VIP}\cdot (\left[{P1C1}_{V}\right]+\left[{P1C2}_{V}\right]+\left[{P2C1}_{V}\right]+\left[{P2C2}_{V}\right])-\ell_{VIP}\cdot \left[VIP\right],\\ {}[6pt]\frac{d\left[AVP\right]}{dt}= & \;k_{AVP}\cdot (\left[{P1C1}_{D}\right]+\left[{P1C2}_{D}\right]+\left[{P2C1}_{D}\right]+\left[{P2C2}_{D}\right])-\ell_{AVP}\cdot \left[AVP\right]. \end{array}$$

The resulting peptide signals were then incorporated into the *Per1* and *Per2* equations of the ventral and dorsal compartments to represent peptide-mediated coupling between SCN subregions. Using the activating and inhibitory Hill functions defined above, the clock gene equations can be written in the following generic form for X∈{V,D}, where *V* stands for ventral SCN and *D* for dorsal SCN:


$$\begin{array}{cc} \frac{d\left[m_{Per1}^{X}\right]}{dt}= & \left(V_{0,P1}+V_{1,P1}g_{\alpha 1,P1}\left([{BC}^{X}]\right)\right)f_{I1,P1}\left([{P1C1}^{X}]\right)f_{I2,P1}\left([{P1C2}^{X}]\right)\\ & f_{I3,P1}\left([{P2C1}^{X}]\right)f_{I4,P1}\left([{P2C2}^{X}]\right)-k_{m,P1}\left[m_{Per1}^{X}\right]+C_{P1}^{X},\;X\in \{V,D\}.\\ {}[6pt]\frac{d\left[m_{Per2}^{X}\right]}{dt}= & \left(V_{0,P2}+V_{1,P2}g_{\alpha 1,P2}\left([{BC}^{X}]\right)\right)f_{I1,P2}\left([{P1C1}^{X}]\right)f_{I2,P2}\left([{P1C2}^{X}]\right)\\ & f_{I3,P2}\left([{P2C1}^{X}]\right)f_{I4,P2}\left([{P2C2}^{X}]\right)-k_{m,P2}\left[m_{Per2}^{X}\right]\\ & +V_{2,HSF1_{3}}g_{3,HSF1_{3}}\left([{HSF1}_{3}^{X}]\right)+C_{P2}^{X},\;X\in \{V,D\}. \end{array}$$

Here, $C_{P1}^{X}$ and $C_{P2}^{X}$ denote the peptide-mediated coupling terms. In the ventral compartment, only VIP-dependent regulation is included, whereas in the dorsal compartment both VIP and AVP-dependent regulation are present. Therefore,


$$\begin{array}{cc} C_{P1}^{V}= & V_{VIP,P1}g_{VIP,P1}\left(\left[{VIP}^{V}\right]\right),\\ {}[6pt]C_{P1}^{D}= & V_{VIP,P1}g_{VIP,P1}\left(\left[{VIP}^{V}\right]\right)+V_{AVP,P1}g_{AVP,P1}\left(\left[{AVP}^{D}\right]\right), \end{array}$$

and $C_{P2}^{V},C_{P2}^{D}$ have a similar form.

The VIP- and AVP-related coupling parameters in the compartmentalized SCN model were also introduced as effective phenomenological terms to represent neuropeptidergic regulation at the model level, rather than as directly measured kinetic constants. In this formulation, VIP provides the dominant coupling signal from the ventral compartment to the SCN network, whereas AVP primarily captures local regulation within the dorsal compartment. Under a constant temperature of 37°C, the model produces a 23.6-h autonomous oscillation and an 8.6-hour phase difference between the ventral and dorsal circadian oscillators, consistent with experimental observations showing that PER2 peak fluorescence occurs earlier in the dorsal SCN than in the ventral SCN [39]. This phase offset is used to represent the intrinsic regional timing difference within the SCN. To compare simulations with experimental measurements, which typically report clock gene expression at the level of the global SCN, we defined the global SCN signal as a weighted average of the ventral and dorsal oscillations. Accordingly, the global SCN signal was taken as one third of the ventral expression plus two thirds of the dorsal expression, based on the approximate 2:1 ratio of AVP to VIP neurons [10,40]. S2 Fig shows representative autonomous dynamics of the compartmentalized SCN model at a constant 37°C, including the mRNA time courses of the core clock genes *Bmal1*, *Per1*, *Per2*, *Cry1*, *Cry2*, and *Rev-erb*α. Additional details of the compartmentalized SCN model, including the full equations, parameter values, and initial conditions, are provided in Section A of S1 Text. To examine the sensitivity of the model behavior to the newly introduced coupling parameters, we performed a Latin hypercube sampling analysis over all such parameters, including those governing both HSF1–clock coupling and VIP/AVP-mediated coupling (Section B of S1 Text). Specifically, 10,000 parameter sets were generated by varying each parameter within ±20% of its nominal value.

## Results

### The model captures distinct thermal responses in peripheral clocks and the SCN

We first tested whether the model could recapitulate the experimentally observed differences in thermal responses between peripheral clocks and the SCN [21]. We began by evaluating the response of the homogeneous model, which serves as an effective representation of peripheral clocks, to acute thermal stimulation through comparison with experimental observations [41]. Consistent with experimental measurements [34], the peak of simulated *Per2* expression was assigned to CT9. We then simulated a 2-hour heat pulse at CT4, during which the temperature was elevated from 37°C to 41°C (gray shading), as shown in Fig 2A. The heat pulse induces a rapid increase in HSF1, which affects *Per2* expression through two opposing pathways. On the one hand, the model includes an inhibitory effect of HBC formation on BMAL1:CLOCK-mediated *E-box* activity, which counteracts *Per2* transcription. On the other hand, elevated HSF1 trimer levels (red) enhance HSE-mediated activation of the *Per2* gene and promote its transcription. The net effect of these competing processes is an acute increase in *Per2* mRNA (dark blue). Thus, the model captures the transient induction of *Per2* in response to temperature elevation. We then compared the magnitude of simulated *Per2* induction with the experimental values reported by Ohnishi et al. [41] to assess whether the model response is quantitatively reasonable. Experimentally, mice were exposed to a 2-hour heat treatment at 41°C starting at CT4. *Per2* mRNA levels in kidney, liver, and submandibular gland were measured relative to the 37°C control condition, which was normalized to 1, allowing the percentage increase induced by heat to be compared across tissues. Under this normalization, the magnitude of acute *Per2* induction in the model falls within the experimentally observed range (Fig 2B), supporting the quantitative reasonableness of the short-term thermal response of peripheral clocks.

**Fig 2:**
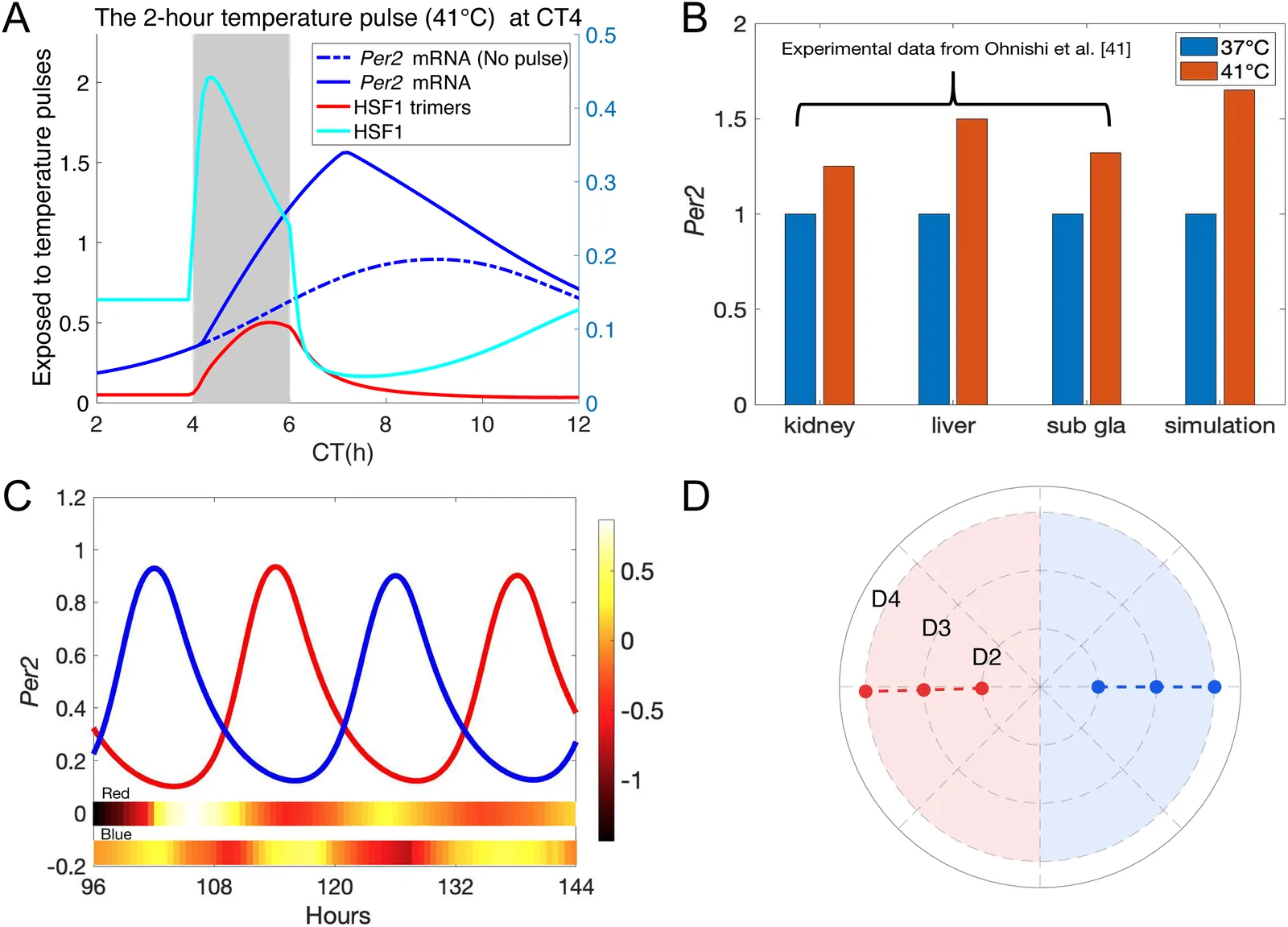
Model and experimental validation of acute and cyclic temperature responses in peripheral clocks. **A**: Model simulation of a 2-hour heat pulse (41°C, shaded gray) applied at CT4, showing *Per2* mRNA (solid line), *Per2* mRNA without heat pulse (dashed line), HSF1 trimers, and HSF1. The heat pulse at CT4 induces a marked acute increase in *Per2* mRNA compared to the no-pulse condition. **B**: Quantitative comparison between experimental values reported by Ohnishi et al. [41] for different peripheral tissues and the simulated fold-change in *Per2* mRNA expression following a 2-hour temperature elevation (41°C vs. 37°C). **C**: Entrainment of peripheral clock to opposing (inverted) temperature cycles. The top shows simulated *Per2* oscillations synchronized to antiphase temperature cycles (36°C/38.5°C). The bottom shows an author-generated heatmap visualization of the experimental data reported by Buhr et al. [21]. **D**: Polar plot of the simulated *Per2* peak phases on days 2, 3, and 4 after entrainment under opposite temperature cycles, shown as three concentric circles from inner to outer. Each dot marks a *Per2* peak phase; blue and red dots correspond to the peak phases of the blue and red curves in Fig 2C, respectively.

Having established that the homogeneous model captures the acute response of peripheral clocks to heat pulses, we next examined whether it could also reproduce entrainment by temperature cycles. Ethan D. Buhr et al. applied the same temperature cycle protocol separately to pituitary and lung explant cultures [21]. In each tissue type, cultures were first synchronized under temperature cycles with the same phase, after which one group was switched to a fully inverted 12-hour temperature cycle between 36°C and 38.5°C while the other remained on the original schedule. After 4 days, *Per2* bioluminescence in the two groups became nearly 180° out of phase in both tissues. For comparison with the model output, the experimental data reported by Buhr et al. [21] were independently visualized in heatmap format. The resulting heatmaps, shown in the bottom of Fig 2C, reveal clear antiphase expression under the two temperature schedules. We then performed corresponding simulations using identical initial conditions and imposed opposite 4-day temperature cycles through the temperature input function *I*. After 4 days of entrainment, the simulated *Per2* oscillations also became entrained to the respective thermal schedules and ultimately evolved into opposite phases (blue and red curves in Fig 2C). Fig 2D further summarizes the simulated *Per2* peak phases after entrainment under opposite temperature cycles. The two entrained states were approximately anti-phasic, consistent with experimental observations in lung and pituitary cultures exposed to inverted schedules [21]. This agreement indicates that the homogeneous model captures the main qualitative outcome of temperature-driven entrainment in peripheral clocks.

Using the same protocol, we next applied a 4-day inverted temperature cycle to the compartmentalized SCN model. In contrast to the homogeneous model, the SCN exhibited only minor phase modulation under opposite temperature schedules and failed to develop antiphase *Per2* rhythms. As shown in Fig 3A, the phase difference between the two conditions remained far smaller than the near 180° separation observed in peripheral clocks. This result is consistent with experimental evidence that the intact SCN is resistant to temperature entrainment [21]; the lower panel of Fig 3A shows the corresponding experimental data reported by Buhr et al. [21], independently visualized here in heatmap format. A natural question is whether this resistance results from weak thermal responsiveness of the SCN. To test this possibility, we examined the model response to a single heat pulse at 38.5°C. As shown in Fig 3B, the global SCN *Per2* level still exhibited a marked acute increase following thermal stimulation. Thus, the failure of the SCN to undergo phase inversion under temperature cycles cannot be explained by an absence of acute heat responsiveness.

**Fig 3:**
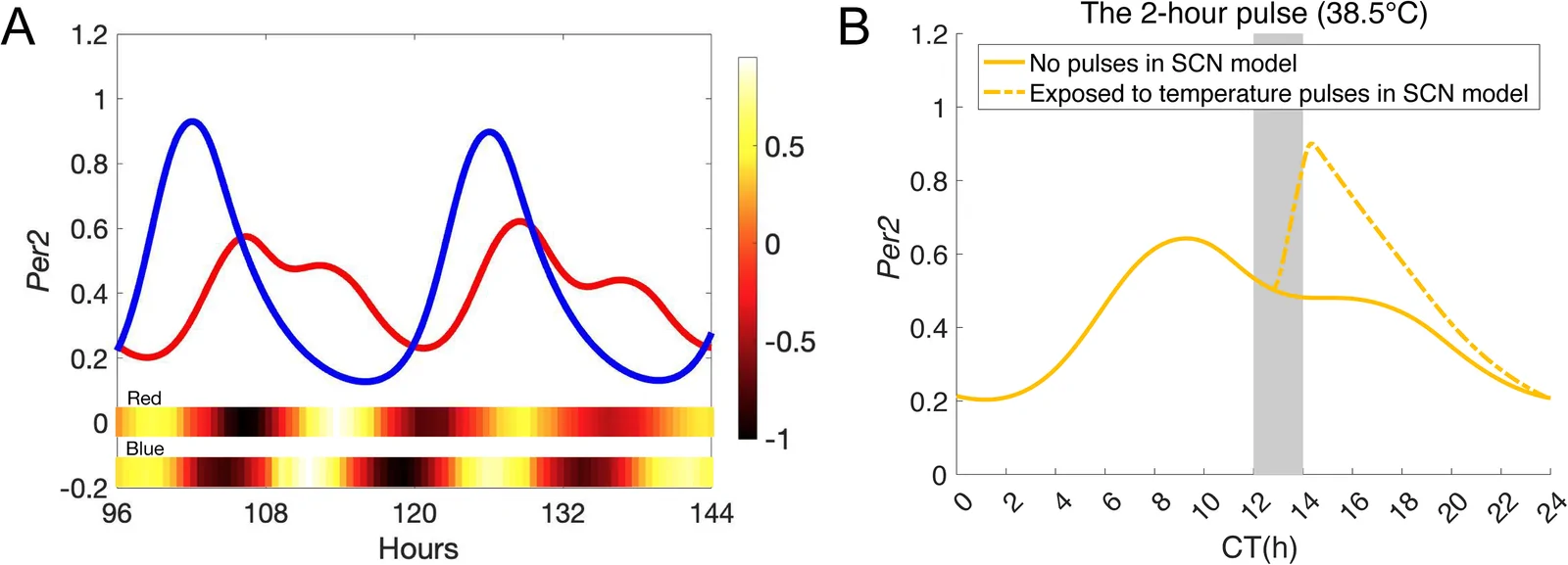
Responses of the SCN model to temperature cycles and heat pulse. **A**: *Per2* rhythms (blue and red lines) do not exhibit phase reversal in response to opposite temperature schedules. The lower heatmaps are author-generated visualizations of the experimental data reported by Buhr et al. [21]. **B**: *Per2* expression in the SCN model in response to a 2-hour heat pulse (38.5°C, gray shaded area) at CT12 after 12-hour low temperature (36°C). The solid and dashed lines represent the control and heat pulse conditions, respectively.

These results establish that the model captures the key experimental contrast between peripheral clocks and the intact SCN under thermal stimulation, providing the basis for subsequent analysis of the mechanism underlying SCN temperature resistance.

### Regional phase differences underlie SCN resistance to thermal entrainment

Because the temperature signaling cascade and the core circadian regulatory network are conserved between the SCN and peripheral tissues, the difference in thermal entrainment is likely to arise from how these signals are integrated within the SCN network. We therefore turned to the spatial organization of the SCN to investigate how internal phase heterogeneity shapes the collective response to thermal input. To dissect this mechanism, we first considered the response of the SCN to a single heat pulse.

The SCN *Per2* phase response was quantified as the shift in the first trough occurring 12 hours after the end of the heat pulse. When a 2-hour temperature pulse at T = 39.1°C was applied at CT6, the global SCN *Per2* exhibited a delay of approximately 1 hour (upper panel, Fig 4A). Because the global SCN signal reflects the combined activity of ventral and dorsal SCN subregions, we next analyzed the responses of these two compartments separately. As shown in the lower panel, the ventral SCN exhibited an approximately 4-hour phase advance in *Per2* (red curve), whereas the dorsal SCN showed an approximately 2.4-hour phase delay (blue curve). Thus, although each subregion displayed a relatively large phase shift in response to the same heat pulse, the two shifts occurred in opposite directions. Since the global SCN *Per2* signal is a weighted integration of ventral and dorsal oscillations, these opposing responses partially cancel at the tissue level, resulting in only a modest net delay of the overall SCN. A similar pattern was observed when the heat pulse was applied at CT18. In this case, the global SCN rhythm exhibited a net phase delay of only approximately 0.3 hours (Fig 4B).

**Fig 4:**
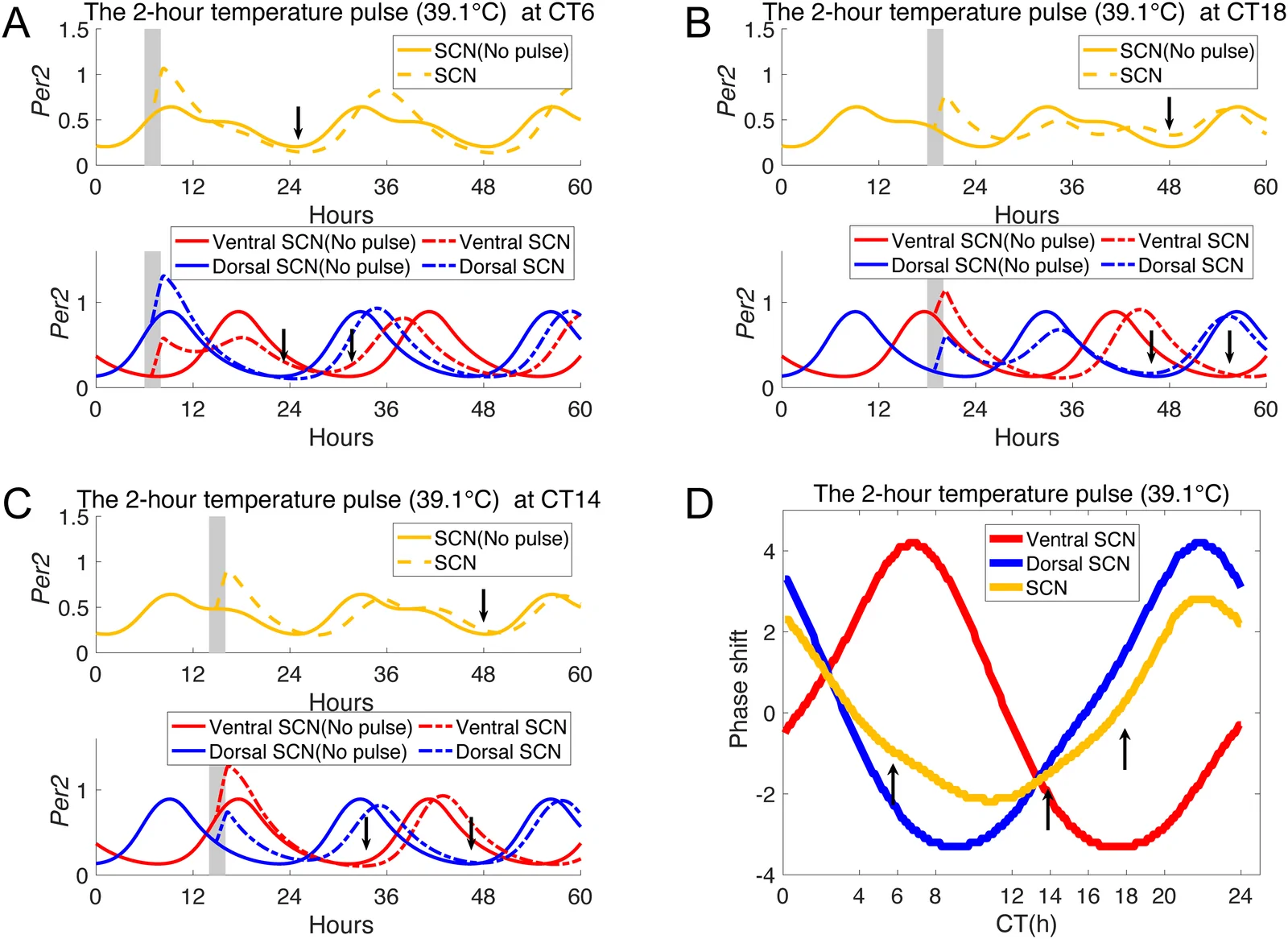
Phase response of *Per2* in the SCN model to temperature pulses. **A–C**: *Per2* expression in the SCN model following a 2-hour temperature pulse (39.1°C, gray shaded) applied at CT6 **(A)**, CT18 **(B)**, or CT14 **(C)**. Upper: Overall SCN *Per2* with and without pulse. Lower: Ventral and dorsal SCN compartments under pulse (solid) and control (dashed). Arrows indicate phase shift measurement. **D**: Phase response curves (PRCs) for ventral, dorsal, and overall SCN to a 2-hour temperature pulse (39.1°C) at different circadian times. Black arrows mark pulse application times at CT6, CT14, and CT18.

At the subregional level, however, the ventral SCN showed a phase delay of about 3.3 hours, whereas the dorsal SCN exhibited a phase advance of about 1.6 hours. Thus, as at CT6, the two compartments responded in opposite directions, and their effects partially canceled in the global SCN signal. Therefore, the responses at CT6 and CT18 illustrate a compensatory mechanism that stabilizes the SCN phase against thermal perturbation. At both stimulation times, the same heat pulse drives opposite phase shifts in the ventral and dorsal SCN, with one region advancing while the other delays. Because the global SCN rhythm integrates signals from both compartments, these opposing responses reduce the net phase shift of the intact SCN.

Although opposing phase responses in the two subregions do not occur at all stimulation times, attenuation of the global SCN phase response remains a general feature across circadian time. For example, when a temperature pulse was applied at CT14 (Fig 4C), both the ventral and dorsal SCN exhibited phase delays of approximately 2.1 and 1.2 hours, respectively, rather than shifting in opposite directions. The global SCN *Per2* therefore showed a net phase delay of about 1.5 hours. However, over the full 24-hour cycle, comparison of the ventral, dorsal, and global SCN *Per2* PRCs shows that the global SCN typically exhibits attenuated phase shifts relative to the subregional responses (Fig 4D). This indicates that interregional compensation dampens net SCN phase resetting even when the two subregions do not respond in strictly opposite directions, thereby contributing to the thermal robustness of the intact SCN.

Since the global SCN signal is constructed as a weighted average of the ventral and dorsal signals, the effect of the assumed ventral:dorsal weighting ratio on this attenuation was examined next. We tested ventral:dorsal weighting ratios of 1:3, 1:1, and 3:2 around the biologically motivated baseline. The resulting global SCN phase shifts were then compared with the corresponding phase shifts of the dorsal and ventral SCN subregions. Across all tested weighting ratios, the above conclusion remained unchanged: the phase shift of the combined global SCN signal lay between the individual dorsal and ventral phase shifts, consistent with a reduced net phase response arising from interregional phase heterogeneity (S3A Fig). Thus, the proposed mechanism does not depend on the specific baseline averaging assumption, although the weighting ratio does influence the extent of this reduction in the global phase response to some degree. As shown in S3A Fig, when the relative contribution of the ventral SCN subregion increases, the phase-cancellation effect becomes more pronounced. S3B and S3C Fig support this trend more directly at two representative stimulus times, CT8 and CT20, where the dorsal and ventral SCN subregions shift in opposite directions. At CT8, increasing the relative ventral contribution progressively reduced the global phase delay, whereas at CT20 it progressively reduced the global phase advance. Thus, despite quantitative differences, the qualitative inference that interregional phase heterogeneity reduces the net global phase shift remains unchanged across weighting ratios.

Having established that the effect was not specific to the weighting ratio, we next asked whether the same qualitative result remained robust to perturbations of the newly introduced coupling parameters. Using Latin hypercube sampling, 10,000 parameter sets were generated by varying all newly introduced parameters within ±20% of their nominal values (see Section B of S1 Text). Among these, 3523 parameter sets produced stable global oscillations with a period near 23.6 h, although the interregional phase difference varied substantially, as shown in S4 Fig. In the majority of synchronized cases, the dorsal SCN led the ventral SCN by up to 11.8 h (*n* = 3201), with most phase differences clustered near 8.5–9 h, whereas a smaller subset showed ventral-leading phase relationships of approximately 6–11.8 h (*n* = 322). Even under these perturbations, the same qualitative result was observed in both lead-lag configurations: whenever a phase difference existed between the two subregions, the global SCN phase shift generally lay between the corresponding ventral and dorsal shifts across stimulus times, consistent with attenuation at the level of the integrated SCN signal (S5 Fig). The same qualitative result was reproduced under a narrower ±10% perturbation range (S6 Fig). Therefore, we conclude that the reduced global SCN phase response emerges as a robust consequence of heterogeneous subregional phase shifts rather than a byproduct of the averaging assumption or a narrowly tuned parameter set.

### SCN thermal resistance decreases as the dorsal–ventral phase difference narrows

Given the attenuation of the global SCN PRC under the natural 8.6-hour phase difference between ventral and dorsal subregions, we next asked whether the magnitude of this phase difference affects the thermal robustness of the SCN. To address this, we systematically varied the phase difference between the two compartments and quantified the resulting global phase shift under thermal stimulation.

To focus specifically on the role of phase difference, we decoupled the ventral and dorsal SCN compartments and adjusted their initial conditions to produce different phase differences between the two subregions. This setup allowed us to directly evaluate how the magnitude of the dorsal–ventral phase difference affects the net phase shift of the SCN in response to heat stimulation. As shown in Fig 5A, when a 2-hour heat pulse was applied at CT6.5 under a dorsal–ventral phase difference of 1 hour, the *Per2* rhythms in the ventral and dorsal SCN were delayed by approximately 2.1 and 2.8 hours, respectively. The resulting global SCN phase delay was about 2.6 hours, which is close to the shifts observed in the individual subregions. In contrast to the natural 8.6-hour phase difference, a 1-hour phase offset produces much less cancellation between regional responses. Thus, when the interregional phase difference is small, the global SCN no longer exhibits strong attenuation of the thermal phase shift. This pattern is also evident in the phase response curves. As shown in Fig 5B, the PRCs of the global SCN (yellow), ventral SCN (red), and dorsal SCN (blue) are highly similar across the tested stimulation times.

**Fig 5:**
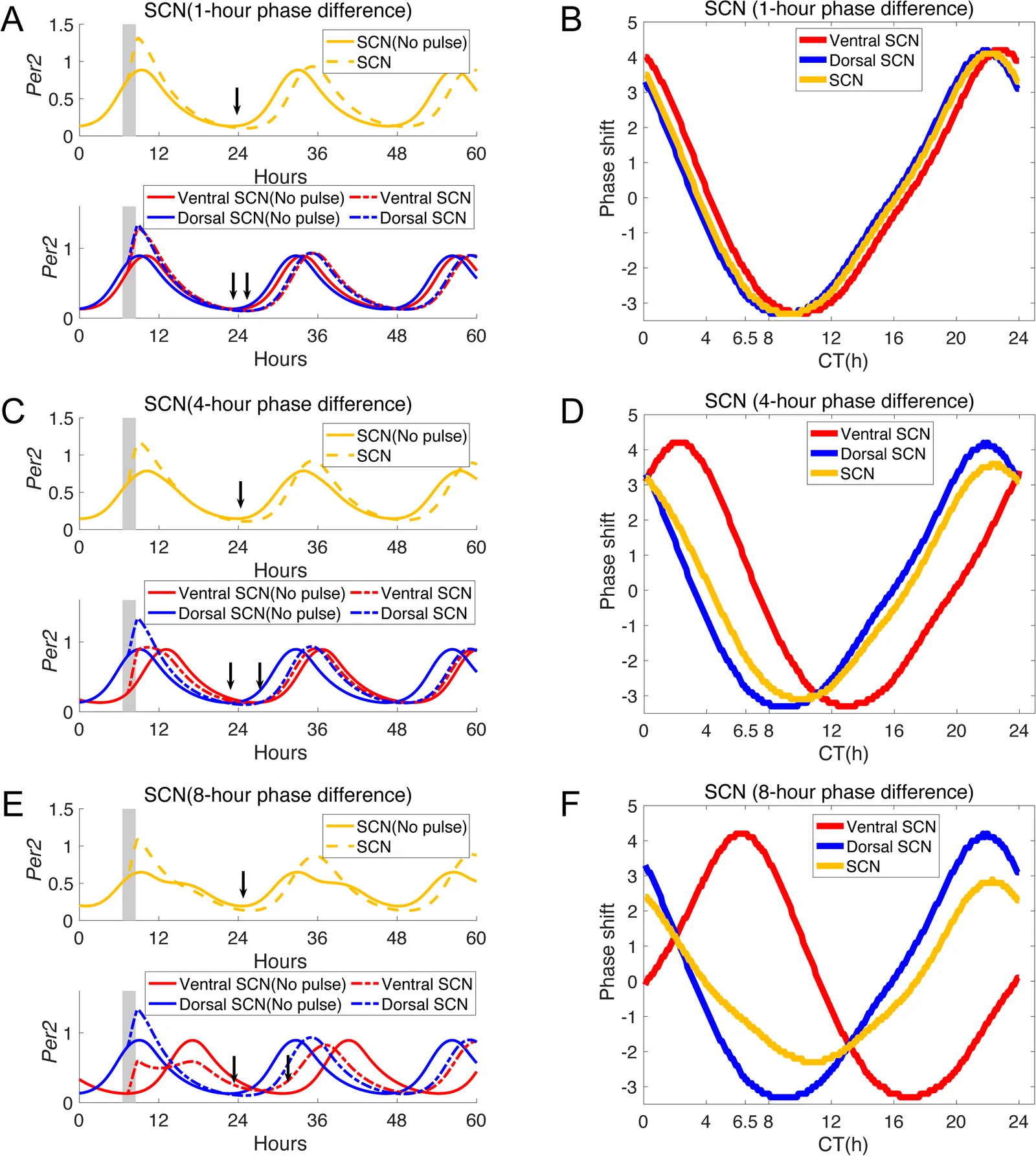
Phase response of the SCN model without VIP coupling for different dorsal–ventral phase differences. **A,B**: *Per2* expression and phase response after a 2-hour, 39.1°C temperature pulse at CT6.5, with a 1-hour phase difference between dorsal and ventral SCN compartments. **(A)** Upper: overall SCN *Per2* expression with and without pulse. Bottom: *Per2* in ventral and dorsal compartments. **(B)** Corresponding PRCs for overall SCN (yellow), ventral (red), and dorsal (blue); the black line indicates the pulse time. The net phase shift of the overall SCN is 2.6 hours. **C,D**: Same as **(A, B)**, but with a 4-hour dorsal–ventral phase difference. The net phase shift of the overall SCN is 1.9 hours. **E,F**: Same as above, but with an 8-hour phase difference. The net phase shift of the overall SCN is 1.1 hours. Solid and dashed lines represent simulations with and without the heat pulse. Arrows indicate measurement of phase shifts.

When the phase difference between ventral and dorsal SCN subregions was increased to 4 hours, the *Per2* rhythm in the ventral SCN advanced by approximately 0.8 hours, whereas the dorsal SCN delayed by approximately 2.8 hours (Fig 5C). Under this condition, the net phase delay of the global SCN was reduced to about 1.9 hours, compared with 2.6 hours when the interregional phase difference was 1 hour. This reduction was also reflected in the global SCN PRC, whose amplitude decreased as the phase difference widened (Fig 5D). A similar trend was observed as the interregional phase difference increased further. When the phase difference was further increased to 8 hours (Fig 5E and 5F), the offset between ventral and dorsal responses became even more pronounced, and the net phase shift of the global SCN decreased to only about 1.1 hours. The amplitude of the global SCN PRC was further reduced under this condition. Thus, increasing the phase difference between ventral and dorsal subregions progressively attenuated the net phase resetting of the SCN.

To quantify this relationship systematically, we varied the phase difference between ventral and dorsal SCN subregions from 0 to 8 h in 1-h increments and measured the resulting global *Per2* phase shift in response to a 2-hour temperature pulse applied at CT6.5 (Fig 6A). The global phase shift decreased as the interregional phase difference increased and reached a minimum at approximately 8 hours. Notably, this minimum occurs close to the natural phase difference used in the SCN model. These results show that larger dorsal–ventral phase differences produce stronger compensation between subregional responses and thereby enhance thermal resistance of the SCN. This dependence on phase difference extends across the circadian cycle. Fig 6B shows a heatmap of the global SCN phase shift over a range of interregional phase and pulse timings. At any given stimulation time, the SCN phase shift becomes smaller as the phase difference between ventral and dorsal subregions increases. In addition, the maximum phase shift observed across all stimulation times is progressively reduced at larger phase differences. These results indicate that the phase difference between ventral and dorsal SCN subregions is a key determinant of SCN thermal resistance, with larger phase differences producing stronger attenuation of temperature-induced phase shifts.

**Fig 6:**
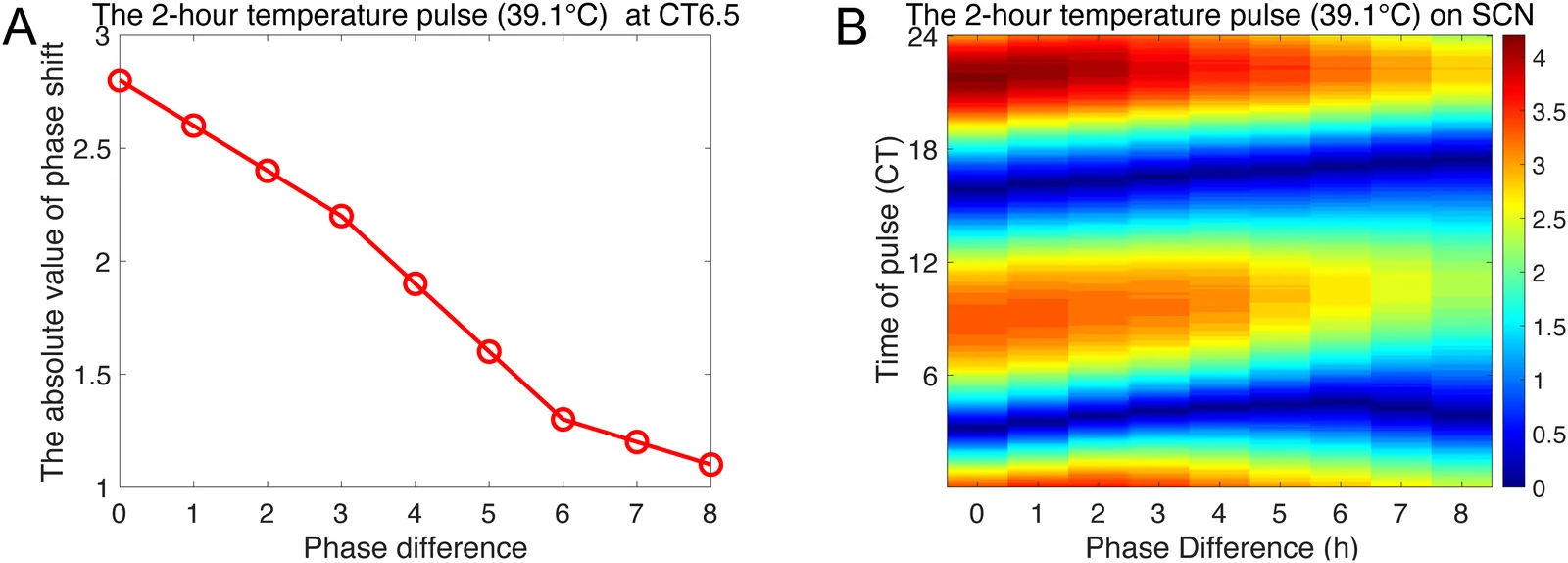
Phase shifts in the SCN decrease as dorsal–ventral phase difference increases. **A**: Absolute value of the *Per2* phase shift in the overall SCN following a 2-hour, 39.1°C temperature pulse applied at CT6.5, shown as a function of dorsal–ventral phase difference. **B**: Heatmap showing the absolute value of *Per2* phase shifts in the SCN after a 2-hour, 39.1°C temperature pulse, plotted across a range of dorsal–ventral phase differences (0–8 hours) and pulse times (CT0–24).

Consistent with this trend, we further analyzed the synchronized parameter sets obtained under the ±20% perturbation range. Cases with relatively small dorsal–ventral phase differences (0.1–4 h; *n* = 70) exhibited larger global SCN phase shifts than most other synchronized cases (S7 Fig), reinforcing the conclusion that when the dorsal and ventral subregions undergo more similar phase shifts, attenuation at the global level is weakened. Thus, both the decoupled phase-difference analysis and the parameter-perturbation analysis support a negative relationship between the magnitude of the dorsal–ventral phase difference and the phase-shift response to thermal stimulation.

### The spatial pattern of thermal input may contribute to SCN thermal resistance

The analyses above showed that the phase difference between the dorsal and ventral SCN can cause the two regions to exhibit different phase shifts in response to the same thermal perturbation, thereby partially canceling the net phase shift of the global SCN rhythm. To further demonstrate that this effect arises primarily from interregional phase heterogeneity, we introduced a reduced two-oscillator Poincaré model. This simplified model retains only the basic dynamical ingredients relevant to the present question, including a stable phase difference between the two oscillators, interregional coupling, and external perturbation. Despite its simplicity, it reproduced the core mechanism identified above: two oscillators at different phases can respond differently to the same perturbation, thereby attenuating the net phase shift of their combined signal (S8 Fig).

Building on this result, we next asked whether the spatial pattern of stimulation also affects how the local phase responses combine into a net phase shift. In the Poincaré model, the phase response curves of the combined signal differed between dual stimulation, in which both oscillators were perturbed simultaneously, and ventral-only stimulation, in which only the ventral-like oscillator was perturbed (Fig 7A). Over approximately half of the circadian cycle, the global phase shift was smaller under dual stimulation than under ventral-only stimulation, as indicated by the shaded region. Notably, this reduced global phase shift occurred under a stronger input condition, because both oscillators received the same perturbation under dual stimulation. Instead, the shaded interval corresponded to phases at which the dorsal-like and ventral-like oscillators shifted in opposite directions in response to the same perturbation, thereby reducing the net phase shift of the combined signal. Representative examples further illustrated this relationship (Fig 7B): under dual stimulation (solid bars), the two oscillators reset in opposite directions, resulting in a reduced net phase shift of the combined signal, whereas ventral-only stimulation (hatched bars) produced a larger global phase shift. The examples include both configurations, with the dorsal-like oscillator delaying while the ventral-like oscillator advanced, and vice versa. We next examined whether the molecular compartmentalized SCN model showed the same pattern as the reduced model. Phase-shift summaries at CT4 and CT18 showed that, under dual stimulation, the dorsal and ventral regions again shifted in opposite directions, with a smaller resulting global phase shift than under ventral-only stimulation (Fig 7B, solid versus hatched bars). The phase response curves of the compartmentalized SCN model were also consistent with this interpretation (S9 Fig). In particular, the phase interval over which dual stimulation produced an attenuated global phase shift fell within the range in which the dorsal and ventral regions shifted in opposite directions, although this interval was relatively narrow. This likely reflects the simplified structure of the present compartmentalized molecular SCN model, in which the global output is represented as a weighted sum of the oscillatory signals from the two subregions.

**Fig 7:**
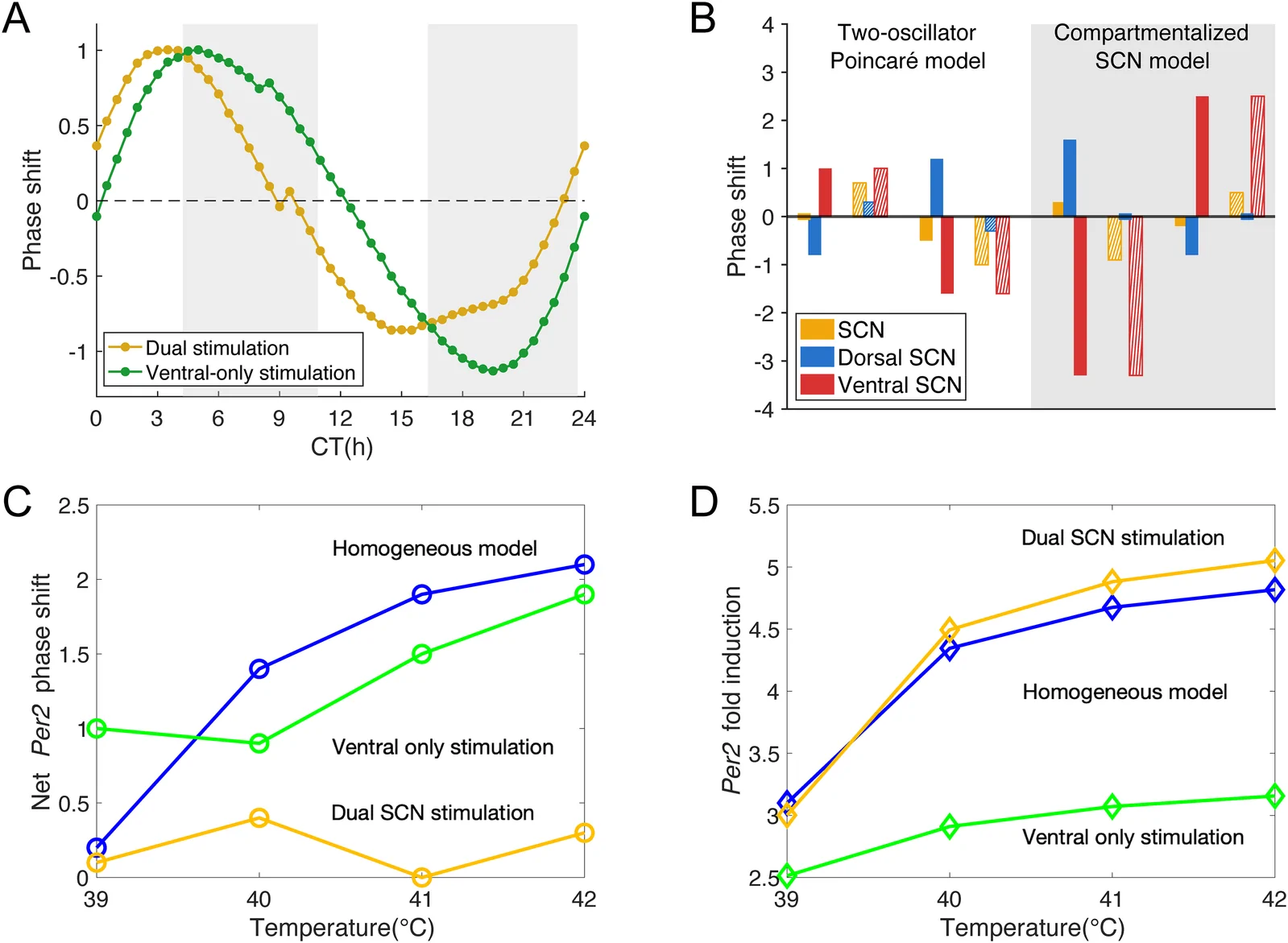
Effect of stimulation pattern on SCN phase shift and *Per2* induction. **A**: Phase response curves of the combined signal in the reduced Poincaré model for ventral-only stimulation (green) and simultaneous stimulation of both oscillators (yellow). Gray shading indicates phases where simultaneous stimulation produces a smaller phase shift than ventral-only stimulation. **B**: Representative phase-shift decompositions are shown under four conditions, corresponding from left to right to Poincaré CT9, Poincaré CT21, molecular CT18, and molecular CT4. Within each condition, solid bars represent dual stimulation and hatched bars represent ventral-only stimulation. Yellow, blue, and red bars indicate the phase shifts of the global SCN signal, dorsal, and ventral components, respectively. **C**: Net *Per2* phase shift after a 2-hour temperature pulse at CT4, as a function of pulse temperature, for three conditions: dual SCN stimulation (yellow), ventral-only stimulation (green), and homogeneous model (blue). **D**: Fold increase in *Per2* expression after stimulation under the same conditions. Fold increase was calculated as the ratio of the maximum *Per2* level after stimulation to the level immediately before stimulation.

We next increased the thermal stimulus at CT4 and compared the responses of the molecular model under dual and ventral-only stimulation together with those of the homogeneous single-oscillator model (Fig 7C and 7D). Even under the stronger perturbation, dual stimulation still produced the smallest phase shift (yellow), whereas the homogeneous model (blue) showed a substantially larger shift. Notably, under dual stimulation, increasing the stimulus strength led to a larger acute increase in *Per2* without producing a larger phase shift, which instead became slightly smaller at 41°C (Fig 7C and 7D). By contrast, under ventral-only stimulation (green) and in the homogeneous model (blue), stronger thermal input increased both the acute *Per2* response and the phase shift, and the phase shifts remained larger than those under dual stimulation. The same qualitative conclusion was also obtained when the acute response was quantified using the absolute *Per2* increase rather than fold induction (S10 Fig).

Thus, in the presence of interregional phase heterogeneity, dual stimulation can produce a substantial acute *Per2* response while limiting the shift of the global circadian phase at certain circadian phases, and this effect can persist even when the thermal stimulus is strengthened.

## Discussion

Many previous circadian models have emphasized detailed molecular pathways to account for temperature compensation, with a primary focus on how circadian period remains stable across changes in ambient temperature [42,43]. By contrast, our study addresses a different aspect of thermal robustness, namely how the SCN resists temperature-induced phase resetting despite receiving thermally responsive input. Using a compartmentalized model of the SCN, we identify population-level organization as one possible source of thermal resistance beyond molecular insensitivity alone. In particular, coupling between ventral and dorsal SCN subregions with distinct intrinsic phases attenuates net phase shifts through interregional compensation. Our results further suggest that the spatial pattern of input delivery also contributes to robustness. At certain phases, simultaneous activation of both subregions produces a substantial acute increase in activity while inducing less net phase resetting than more spatially restricted stimulation. These findings highlight SCN architecture, including both internal phase structure and stimulation pattern, as an important contributor to functional stability under thermal perturbation. This view is consistent with previous modeling studies suggesting that SCN network structure shapes collective phase organization and thereby regulates sensitivity to light input [44]. Our results extend this network-level perspective to thermal phase resetting and suggest that even a minimal two-compartment framework can capture aspects of SCN organization that would be missed in purely single-cell descriptions. Moreover, our work focuses on thermal input, which acts concurrently on both SCN regions, whereas photic cues are believed to act primarily on the ventral SCN. These differences in input pattern and network context suggest that distinct mechanisms underlie resistance to thermal versus photic entrainment.

Beyond the two-compartment phase organization described above, several additional factors are also known to influence phase resetting behavior. It is therefore important to clarify how the mechanism identified here relates to them. Oscillation amplitude, for instance, can modulate phase responsiveness through at least two distinct routes: for a single oscillator, reduced amplitude can increase susceptibility to perturbation and bring the system closer to a singularity-like resetting regime [45], whereas at the population level, reduced amplitude may instead reflect weakened coupling and loss of coherence across oscillators, so that perturbation-induced shifts in the mean phase are less effectively restored [46]. The mechanism identified here is distinct from amplitude-driven susceptibility and from coupling-mediated stabilization. Attenuation of the global SCN phase response in our model arises primarily because the same thermal stimulus induces different phase shifts in the two SCN subregions, which then partially cancel in the global SCN phase. These other factors, meanwhile, are not mutually exclusive. This becomes particularly relevant when comparing our model with the experimental results of Buhr et al. [21], where even prolonged heat stimulation produced only a ~0.3 h phase shift in the intact SCN. In contrast, with a 2-h thermal stimulus, our model, which incorporates phase heterogeneity and simultaneous stimulation of both subregions, still yielded residual shifts exceeding 1 h. The substantially weaker response observed experimentally therefore suggests that these mechanisms alone are unlikely to be sufficient, and that additional stabilizing processes, including stronger within-region coupling, may also contribute in vivo.

Despite these strengths, the present model also highlights several areas for future investigation. First, although the two-compartment formulation captures essential differences between ventral and dorsal SCN regions, the real SCN is a densely connected neuronal network with far more complex coupling topology. By not explicitly representing heterogeneity in coupling strength, nonuniform connectivity, or regional synaptic organization, the model may underestimate how network architecture contributes to resistance against thermal perturbation. Second, our representation of the SCN as two averaged compartments simplifies what is likely a continuous distribution of circadian phases across individual SCN neurons. Such continuous phase organization may be important for fine-tuning responses to environmental input and for maintaining coherence under fluctuating conditions. Compressing this diversity into two populations may therefore omit dynamical features that emerge from broader phase dispersion. This simplification may also affect our analysis of stimulation pattern. In the ventral-only stimulation condition, thermal pulses at some circadian times produced response trajectories with secondary peaks, making the phase shift less straightforward to quantify than in the simultaneous stimulation and homogeneous conditions. Accordingly, the effect of stimulation pattern was illustrated using representative cases rather than analyzed systematically across the full circadian cycle. Third, the model focuses on interregional coupling and heat-shock-mediated clock regulation, but other biological mechanisms are also likely to contribute to thermal robustness. Experimental findings that the SCN can still be entrained by temperature after neuronal coupling is disrupted by TTX indicate that cell-to-cell coupling plays an important role in shaping the phase response to thermal input [21]. In this context, our results point to an additional network-level contribution, namely that phase organization and input integration across SCN subregions can buffer thermal resetting at the population level. Incorporating cell-to-cell coupling and phase heterogeneity into future models may help clarify how these factors jointly regulate phase shifts in response to external stimuli, potentially accounting for a wider range of experimental observations. Finally, another related factor not explicitly considered in the present framework is period heterogeneity across SCN oscillators. Because differences in intrinsic period can also generate or modify phase differences between oscillators [29], period heterogeneity may further influence collective thermal responses and should be examined in future work.

More broadly, future extensions of this framework to more realistic network architectures may provide a useful basis for studying how circuit-level organization shapes circadian adaptation, robustness, and vulnerability under environmental stress.

## Supporting information

S1 Text Supplementary model equations, parameter values, and robustness analyses. S1 Text consists of three sections. Section A presents the equations, parameters, and initial conditions for the peripheral clock and two-compartment SCN models (A1–A6 Tables). Section B describes the parameter robustness analysis using Latin hypercube sampling and summarizes the parameters included in the analysis (B1 Table). Section C introduces the conceptual two-oscillator model based on Poincaré oscillators and provides the corresponding model, stimulation, and numerical parameters (C1 Table).
(PDF)

S1 Fig mRNA expression profiles of circadian genes in the peripheral oscillator model. (PDF)

S2 Fig mRNA expression profiles of circadian genes in the two-compartment SCN model. (PDF)

S3 Fig Effect of ventral–dorsal weighting on the global SCN phase response to a 2-h temperature pulse. (PDF)

S4 Fig Distribution of the signed dorsal-to-ventral SCN phase difference among the synchronized parameter sets in the 20% analysis. (PDF)

S5 FigGlobal and subregional phase responses under ±20% perturbations of the newly introduced parameters.(PDF)

S6 FigGlobal and subregional phase responses under ±10% perturbations of the newly introduced parameters.(PDF)

S7 Fig Subregional and global SCN phase-shift distributions across CT for synchronized parameter sets with small dorsal–ventral phase differences. (PDF)

S8 Fig Phase-response curves in the two-oscillator Poincaré model under simultaneous stimulation of the dorsal-like and ventral-like oscillators. (PDF)

S9 Fig Phase-response curves of the compartmentalized SCN model under different spatial patterns of thermal stimulation. (PDF)

S10 Fig Difference in *Per2* expression after stimulation at different temperatures. (PDF)

S1 Code Source code used to reproduce all main and supplementary figures. (ZIP)
