# Supplementary material for: A compartmentalized SCN model explains temperature resistance through interregional phase differences
Source: PLoS Comput Biol. 2026 Sep 29;22(9):e1014831. doi: 10.1371/journal.pcbi.1014831 (PMC13645230; doi:10.1371/journal.pcbi.1014831)
Supplement: Supplementary file 1 [file pcbi.1014831.s001.pdf]

S1 Text

Contents

A Model Details for the SCN and Peripheral Clocks **2**

1 Equations, Parameters, and Initial Conditions for the Peripheral Clock Model 2

2 Equations, Parameters, and Initial Conditions for the Two-Compartment SCN Model 15

B Robustness Analysis of Parameters Using Latin Hypercube Sampling **24**

C A Conceptual Two-Oscillator Model Based on Poincaré Oscillators **28**

A Model Details for the SCN and Peripheral Clocks

1 Equations, Parameters, and Initial Conditions for the Peripheral Clock Model

We present the full set of 21 ordinary differential equations (Eqs. 1–21) for the mammalian circadian clock, based on the model by Mirsky et al. This set forms the core of the model. In this formulation, activation of *Per2* mRNA by the HSF1 trimer and the interaction between HSF1 and the BMAL1:CLOCK complex link the circadian oscillator to the HSF1–HSP system (see Eqs. 22–31, especially Eq. 23). Since the original HSF1–HSP subsystem uses a minute timescale, all relevant rate constants were multiplied by 60 to convert to an hourly basis for consistency with the circadian model. Additionally, terms abbreviated in the main text as $g(.)$ and $f(.)$ are shown here in expanded form for clarity.

A1 Table lists the variable symbols. A2 Table lists the parameter definitions, symbols, and values. A3 Table lists the remaining parameter definitions, symbols, and values. The ODEs follow.

$$\begin{aligned}\frac{dmPer1}{dt} &= \left(V0_{P1} + \frac{V1_{P1}\cdot BC^{na1\_P1}}{KA1_{P1}^{na1\_P1} + BC^{na1\_P1}}\right)\cdot\frac{KI1_{P1}^{ni1\_P1}}{KI1_{P1}^{ni1\_P1} + P1C1^{ni1\_P1}}\\ &\cdot\frac{KI2_{P1}^{ni2\_P1}}{KI2_{P1}^{ni2\_P1} + P1C2^{ni2\_P1}}\cdot\frac{KI3_{P1}^{ni3\_P1}}{KI3_{P1}^{ni3\_P1} + P2C1^{ni3\_P1}}\\ &\cdot\frac{KI4_{P1}^{ni4\_P1}}{KI4_{P1}^{ni4\_P1} + P2C2^{ni4\_P1}} - km\_P1\cdot mPer1\end{aligned} \tag{1}$$

$$\begin{aligned}\frac{dmPer2}{dt} &= \left(V0_{P2} + \frac{V1_{P2}\cdot BC^{na1\_P2}}{KA1_{P2}^{na1\_P2} + BC^{na1\_P2}}\right)\cdot\frac{KI1_{P2}^{ni1\_P2}}{KI1_{P2}^{ni1\_P2} + P1C1^{ni1\_P2}}\\ &\cdot\frac{KI2_{P2}^{ni2\_P2}}{KI2_{P2}^{ni2\_P2} + P1C2^{ni2\_P2}}\cdot\frac{KI3_{P2}^{ni3\_P2}}{KI3_{P2}^{ni3\_P2} + P2C1^{ni3\_P2}}\\ &\cdot\frac{KI4_{P2}^{ni4\_P2}}{KI4_{P2}^{ni4\_P2} + P2C2^{ni4\_P2}} - km_{P2}\cdot mPer2\\ &+ V2_{hsf1\_3}\cdot\frac{HSF1\_3^{m3\_hsf1\_3}}{K3_{hsf1\_3}^{m3\_hsf1\_3} + HSF1\_3^{m3\_hsf1\_3}}\end{aligned} \tag{2}$$

$$\begin{aligned}\frac{dmCry1}{dt} &= \left(V0_{C1} + \frac{V1_{C1}\cdot BC^{na1\_C1}}{KA1_{C1}^{na1\_C1} + BC^{na1\_C1}} + \frac{V2_{C1}\cdot RORc^{na2\_C1}}{KA2_{C1}^{na2\_C1} + RORc^{na2\_C1}}\right)\\ &\cdot\frac{KI1_{C1}^{ni1\_C1}}{KI1_{C1}^{ni1\_C1} + P1C1^{ni1\_C1}}\cdot\frac{KI2_{C1}^{ni2\_C1}}{KI2_{C1}^{ni2\_C1} + P1C2^{ni2\_C1}}\cdot\frac{KI3_{C1}^{ni3\_C1}}{KI3_{C1}^{ni3\_C1} + P2C1^{ni3\_C1}}\\ &\cdot\frac{KI4_{C1}^{ni4\_C1}}{KI4_{C1}^{ni4\_C1} + P2C2^{ni4\_C1}}\cdot\frac{KI5_{C1}^{ni5\_C1}}{KI5_{C1}^{ni5\_C1} + R\_E^{ni5\_C1}} - km_{C1}\cdot mCry1\end{aligned} \tag{3}$$

$$\frac{dmCry2}{dt} = \left(V0_{C2} + \frac{V1_{C2} \cdot BC^{na1\_C2}}{KA1_{C2}^{na1\_C2} + BC^{na1\_C2}} + \frac{V2_{C2} \cdot RORc^{na2\_C2}}{KA2_{C2}^{na2\_C2} + RORc^{na2\_C2}}\right)$$
$$\cdot \frac{KI1_{C2}^{ni1\_C2}}{KI1_{C2}^{ni1\_C2} + P1C1^{ni1\_C2}} \cdot \frac{KI2_{C2}^{ni2\_C2}}{KI2_{C2}^{ni2\_C2} + P1C2^{ni2\_C2}} \cdot \frac{KI3_{C2}^{ni3\_C2}}{KI3_{C2}^{ni3\_C2} + P2C1^{ni3\_C2}}$$
$$\cdot \frac{KI4_{C2}^{ni4\_C2}}{KI4_{C2}^{ni4\_C2} + P2C2^{ni4\_C2}} \cdot \frac{KI5_{C2}^{ni5\_C2}}{KI5_{C2}^{ni5\_C2} + R\_E^{ni5\_C2}} - km_{C2} \cdot mCry2 \quad (4)$$

$$\frac{dmRev\_erb\alpha}{dt} = \frac{V1_{Re} \cdot BC^{na1\_Re}}{KA1_{Re}^{na\_Re} + BC^{na1\_Re}} \cdot \frac{KI1_{Re}^{ni1\_Re}}{KI1_{Re}^{ni1\_Re} + P1C1^{ni1\_Re}}$$
$$\cdot \frac{KI2_{Re}^{ni2\_Re}}{KI2_{Re}^{ni2\_Re} + P1C2^{ni2\_Re}} \cdot \frac{KI3_{Re}^{ni3\_Re}}{KI3_{Re}^{ni3\_Re} + P2C1^{ni3\_Re}}$$
$$\cdot \frac{KI4_{Re}^{ni4\_Re}}{KI4_{Re}^{ni4\_Re} + P2C2^{ni4\_Re}} - km_{Re} \cdot mRev\_erb\alpha \quad (5)$$

$$\frac{dmClk}{dt} = \left(V0_{Clk} + \frac{V1_{Clk} \cdot RORc^{na1\_Clk}}{KA1_{Clk}^{na1\_Clk} + RORc^{na1\_Clk}}\right) \cdot \left(\frac{KI1_{Clk}^{ni1_{Clk}}}{KI1_{Clk}^{ni1_{Clk}} + R_E^{ni1_{Clk}}}\right)$$
$$- km_{Clk} \cdot mClk \quad (6)$$

$$\frac{dmBmal1}{dt} = \left(V0_{Bmal1} + \frac{V1_{Bmal1} \cdot RORc^{na1\_Bmal1}}{KA1_{Bmal1}^{na1\_Bmal1} + RORc^{na1\_Bmal1}}\right)$$
$$\cdot \frac{KI1_{Bmal1}^{ni1\_Bmal1}}{KI1_{Bmal1}^{ni1\_Bmal1} + R\_E^{ni1\_Bmal1}} - km_{Bmal1} \cdot mBmal1 \quad (7)$$

$$\frac{dmRorc}{dt} = \left(V0_{Rorc} + \frac{V1_{Rorc} \cdot BC^{na1\_Rorc}}{KA1_{Rorc}^{na1\_Rorc} + BC^{na1\_Rorc}} + \frac{V2_{Rorc} \cdot RORc^{na2\_Rorc}}{KA2_{Rorc}^{na2\_Rorc} + RORc^{na2\_Rorc}}\right)$$
$$\cdot \frac{KI1_{Rorc}^{ni1\_Rorc}}{KI1_{Rorc}^{ni1\_Rorc} + P1C1^{ni1\_Rorc}} \cdot \frac{KI2_{Rorc}^{ni2\_Rorc}}{KI2_{Rorc}^{ni2\_Rorc} + P1C2^{ni2\_Rorc}}$$
$$\cdot \frac{KI3_{Rorc}^{ni3\_Rorc}}{KI3_{Rorc}^{ni3\_Rorc} + P2C1^{ni3\_Rorc}} \cdot \frac{KI4_{Rorc}^{ni4\_Rorc}}{KI4_{Rorc}^{ni4\_Rorc} + P2C2^{ni4\_Rorc}}$$
$$\cdot \frac{KI5_{Rorc}^{ni5\_Rorc}}{KI5_{Rorc}^{ni5\_Rorc} + R\_E^{ni5\_Rorc}} - km_{Rorc} \cdot mRorc \quad (8)$$

$$\frac{dPER1}{dt} = t\_P1 \cdot mPer1 - a\_P1C1 \cdot PER1 \cdot CRY1 - a\_P1C2 \cdot PER1 \cdot CRY2$$
$$+ d\_P1C1 \cdot P1C1 + d\_P1C2 \cdot P1C2 - kp\_P1 \cdot PER1 \quad (9)$$

$$\frac{dPER2}{dt} = t\_P2 \cdot mPer2 - a\_P2C1 \cdot PER2 \cdot CRY1 - a\_P2C2 \cdot PER2 \cdot CRY2$$
$$+ d\_P2C1 \cdot P2C1 + d\_P2C2 \cdot P2C2 - kp\_P2 \cdot PER2 \quad (10)$$

$$\frac{dCRY1}{dt} = t\_C1 \cdot mCry1 - a\_P1C1 \cdot PER1 \cdot CRY1 - a\_P2C1 \cdot PER2 \cdot CRY1$$
$$+ d\_P1C1 \cdot P1C1 + d\_P2C1 \cdot P2C1 - kp\_C1 \cdot CRY1 \quad (11)$$

$$\frac{dCRY2}{dt} = t\_C2 \cdot mCry2 - a\_P1C2 \cdot PER1 \cdot CRY2 - a\_P2C2 \cdot PER2 \cdot CRY2$$
$$+ d\_P1C2 \cdot P1C2 + d\_P2C2 \cdot P2C2 - kp\_C2 \cdot CRY2 \quad (12)$$

$$\frac{dREV\_ERB\alpha}{dt} = t_{REV\_ERB} \cdot mRev\_erb\alpha - kp\_REV\_ERB \cdot R\_E \tag{13}$$

$$\frac{dCLK}{dt} = t\_CLK \cdot mClk - a\_CLK\_BMAL1 \cdot CLK \cdot BMAL1$$
$$+ d\_CLK\_BMAL1 \cdot BC - kp\_CLK \cdot CLK \tag{14}$$

$$\frac{dBMAL1}{dt} = t\_Bmal1 \cdot mBmal1 - a\_CLK\_BMAL1 \cdot CLK \cdot BMAL1$$
$$+ d\_CLK\_BMAL1 \cdot BC - kp\_BMAL1 \cdot BMAL1 \tag{15}$$

$$\frac{dRORc}{dt} = t\_Rorc \cdot mRorc - kp\_RORc \cdot RORc \tag{16}$$

$$\frac{dP1C1}{dt} = a\_P1C1 \cdot PER1 \cdot CRY1 - d\_P1C1 \cdot P1C1 \tag{17}$$

$$\frac{dP2C1}{dt} = a\_P2C1 \cdot PER2 \cdot CRY1 - d\_P2C1 \cdot P2C1 \tag{18}$$

$$\frac{dP1C2}{dt} = a\_P1C2 \cdot PER1 \cdot CRY2 - d\_P1C2 \cdot P1C2 \tag{19}$$

$$\frac{dP2C2}{dt} = a\_P2C2 \cdot PER2 \cdot CRY2 - d\_P2C2 \cdot P2C2 \tag{20}$$

$$\frac{dBC}{dt} = a\_CLK\_BMAL1 \cdot CLK \cdot BMAL1 - d\_CLK\_BMAL1 \cdot BC$$
$$- a\_HSF1\_BMAL1\_CLK \cdot BC \cdot HSF1 + d\_HSF1\_BMAL1\_CLK \cdot HBC \tag{21}$$

$$\frac{dHBC}{dt} = a\_HSF1\_BMAL1\_CLK \cdot BC \cdot HSF1$$
$$- d\_HSF1\_BMAL1\_CLK \cdot HBC \tag{22}$$

$$\frac{dHSF1}{dt} = -k1 \cdot hsp \cdot HSF1 + l1 \cdot hsp\_HSF1 - 3 \cdot k3 \cdot HSF1^3 + 2 \cdot l3 \cdot hsp \cdot HSF1\_3$$
$$+ k6 \cdot hsp\_HSF1 \cdot S - l6 \cdot hsp\_S \cdot HSF1 - a\_HSF1\_BMAL1\_CLK \cdot BC \cdot HSF1$$
$$+ d\_HSF1\_BMAL1\_CLK \cdot HBC \tag{23}$$

$$\frac{dhsp}{dt} = -k1 \cdot hsp \cdot HSF1 + l1 \cdot hsp\_HSF1 - k2 \cdot hsp \cdot S + l2 \cdot hsp\_S$$
$$- l3 \cdot hsp \cdot HSF1\_3 + k4 \cdot mhsp + k10 \cdot hsp\_S - l10 \cdot hsp \tag{24}$$

$$\frac{dS}{dt} = l2 \cdot hsp\_S - k2 \cdot hsp \cdot S - k6 \cdot hsp\_HSF1 \cdot S + l6 \cdot hsp\_S \cdot HSF1 + \mathrm{I} \tag{25}$$

$$\frac{dhsp\_HSF1}{dt} = k1 \cdot hsp \cdot HSF1 - l1 \cdot hsp\_HSF1 - k6 \cdot hsp\_HSF1 \cdot S + l6 \cdot hsp\_S \cdot HSF1$$
$$+ l3 \cdot hsp \cdot HSF1\_3 \tag{26}$$

$$\frac{dhsp\_S}{dt} = k2 \cdot hsp \cdot S - l2 \cdot hsp\_S + k6 \cdot hsp\_HSF1 \cdot S - l6 \cdot hsp\_S \cdot HSF1$$
$$- k10 \cdot hsp\_S \tag{27}$$

$$\frac{dHSF1\_3}{dt} = k3 \cdot HSF1^3 - l3 \cdot hsp \cdot HSF1\_3 - k7 \cdot HSF1\_3 \cdot HSE + l7 \cdot HSF1\_3\_HSE \tag{28}$$

$$\frac{dHSE}{dt} = -k7 \cdot HSF1\_3 \cdot HSE + l7 \cdot HSF1\_3\_HSE \tag{29}$$

$$\frac{dHSF1\_3\_HSE}{dt} = k7 \cdot HSF1\_3 \cdot HSE - l7 \cdot HSF1\_3\_HSE \tag{30}$$

$$\frac{dmhsp}{dt} = k8 \cdot HSF1\_3\_HSE - k4 \cdot mhsp \tag{31}$$

The functional form of Temperature can be written as (T represents the ambient temperature):

$$I = k_I \cdot \left[\left(1 - \frac{0.4}{e^{T-37}} \right) \cdot 0.03 \cdot 1.4^{T-37} \right] \tag{32}$$

A1 Table Symbols of the variables for circadian clock model

Symbols	Variables
mPer1	Per1 mRNA
mPer2	Per2 mRNA
mCry1	Cry1 mRNA
mCry2	Cry2 mRNA
mRev_erb α	mRev_erb α mRNA
mClk	Clock mRNA
mBmal1	Bmal1 mRNA
mRorc	Rorc mRNA
PER1	PER1 protein
PER2	PER2 protein
CRY1	CRY1 Protein
CRY2	CRY2 Protein
R_E	REV_ERB α Protein
CLK	CLOCK Protein
mBMAL1	BMAL1 Protein
RORc	RORc Protein
P1C1	PER1/CRY1 complex
P1C2	PER1/CRY2 complex
P2C1	PER2/CRY1 complex

Continued on next page

A1 Table continued

Symbols	Variables
P2C2	PER2/CRY2 complex
BC	CLOCK/BMAL1 complex
HBC	HSF1/BMAL1/CLK complex
HSF1	HSF1 monomer
hsp	hsp Protein
S	a proper substrate(e.g. a denatured protein or a misfolded protein)
hsp_HSF1	hsp_HSF1 complex
hsp_S	hsp and substrate complex
HSF1_3	HSF1 trimer
HSE	a specific DNA recognition sequence
HSF1_3_HSE	HSF trimers bind to HSE
mhsp	hsp mRNA

A2 Table The definitions and values for the model of the mammalian circadian clock

Parameter name	Parameter symbol	Value
Basal rate of mPer1 transcription	$V0_{P1}$	0.17nM/h
Maximum rate of mPer1 transcription from CLK_BMAL1 activation	$V1_{P1}$	3.84nM/h
Basal rate of mPer2 transcription	$V0_{P2}$	0.09nM/h
Maximum rate of mPer2 transcription from CLK_BMAL1 activation	$V1_{P2}$	3.29nM/h
Basal rate of mCry1 transcription	$V0_{C1}$	0.26nM/h
Maximum rate of mCry1 transcription from CLK_BMAL1 activation	$V1_{C1}$	2.44nM/h
Maximum rate of mCry1 transcription from RORc activation	$V2_{C1}$	2.89nM/h

A3 Table The definitions and values for the model of the mammalian circadian clock (continued).

Parameter name	Parameter symbol	Value
Basal rate of mCry2 transcription	$V0_{C2}$	1.29nM/h
Maximum rate of mCry2 transcription from CLK_BMAL1 activation	$V1_{C2}$	2.72nM/h
Maximum rate of mCry2 transcription from RORc activation	$V2_{C2}$	4.28nM/h
Maximum rate of REV_ERB α transcription from CLK_BMAL1 activation	$V1_{Re}$	1.03nM/h
Basal rate of mClk transcription	$V0_{Clk}$	3.98nM/h
Maximum rate of mClk transcription from RORc activation	$V1_{Clk}$	3.36nM/h
Basal rate of mBmal1 transcription	$V0_{Bmal1}$	1.98nM/h
Maximum rate of mBmal1 transcription from RORc activation	$V1_{Bmal1}$	4.12nM/h
Basal rate of mRorc transcription	$V0_{Rorc}$	0.69nM/h
Maximum rate of mRorc transcription from CLK_BMAL1 activation	$V1_{Rorc}$	3.55nM/h
Maximum rate of mRorc transcription from RORc activation	$V2_{Rorc}$	0.46nM/h
Hill coeffificient for mPer1 transcription due to CLK_BMAL1 activation	$na1\_P1$	3.28
Hill coeffificient for mPer1 transcription due to PER1_CRY1 inhibition	$ni1\_P1$	3.93
Hill coeffificient for mPer1 transcription due to PER1_CRY2 inhibition	$ni2\_P1$	2.61
Hill coeffificient for mPer1 transcription due to PER2_CRY1 inhibition	$ni3\_P1$	1.66
Hill coeffificient for mPer1 transcription due to PER2_CRY2 inhibition	$ni4\_P1$	4.85
Hill coeffificient for mPer2 transcription due to CLK_BMAL1 activation	$na1\_P2$	4.50
Hill coeffificient for mPer2 transcription due to PER1_CRY1 inhibition	$ni1\_P2$	4.45
Hill coeffificient for mPer2 transcription due to PER1_CRY2 inhibition	$ni2\_P2$	3.70

Continued on next page

A3 Table continued

Parameter name	Parameter symbol	Value
Hill coeffificient for mPer2 transcription due to PER2_CRY1 inhibition	$ni3\_P2$	4.35
Hill coeffificient for mPer2 transcription due to PER2_CRY2 inhibition	$ni4\_P2$	3.67
Hill coeffificient for mCry1 transcription due to CLK_BMAL1 activation	$na1\_C1$	4.91
Hill coeffificient for mCry1 transcription due to RORc activation	$na2\_C1$	3.01
Hill coeffificient for mCry1 transcription due to PER1_CRY1 inhibition	$ni1\_C1$	4.65
Hill coeffificient for mCry1 transcription due to PER1_CRY2 inhibition	$ni2\_C1$	3.66
Hill coeffificient for mCry1 transcription due to PER2_CRY1 inhibition	$ni3\_C1$	2.59
Hill coeffificient for mCry1 transcription due to PER2_CRY2 inhibition	$ni4\_C1$	0.29
Hill coeffificient for mCry1 transcription due to REV_ERBα inhibition	$ni5\_C1$	2.24
Hill coeffificient for mCry2 transcription due to CLK_BMAL1 activation	$na1\_C2$	4.39
Hill coeffificient for mCry2 transcription due to RORc activation	$na2\_C2$	4.43
Hill coeffificient for mCry2 transcription due to PER1_CRY1 inhibition	$ni1\_C2$	4.68
Hill coeffificient for mCry2 transcription due to PER1_CRY2 inhibition	$ni2\_C2$	4.71
Hill coeffificient for mCry2 transcription due to PER2_CRY1 inhibition	$ni3\_C2$	2.23
Hill coeffificient for mCry2 transcription due to PER2_CRY2 inhibition	$ni4\_C2$	0.48
Hill coeffificient for mCry2 transcription due to REV_ERBα inhibition	$ni5\_C2$	1.75
Hill coeffificient for mRev_erbα transcription due to CLK_BMAL1 activation	$na1\_Re$	4.40
Hill coeffificient for mRev_erbα transcription due to PER1_CRY1 inhibition	$ni1\_Re$	0.30

Continued on next page

A3 Table continued

Parameter name	Parameter symbol	Value
Hill coeffificient for mRev_erbα transcription due to PER1_CRY2 inhibition	$ni2\_Re$	0.67
Hill coeffificient for mRev_erbα transcription due to PER2_CRY1 inhibition	$ni3\_Re$	1.93
Hill coeffificient for mRev_erbα transcription due to PER2_CRY2 inhibition	$ni4\_Re$	3.58
Hill coeffificient for mClk transcription due to RORc activation	$na1\_Clk$	3.50
Hill coeffificient for mClk transcription due to REV_ERBα inhibition	$ni1\_Clk$	1.96
Hill coeffificient for mBmal1 transcription due to RORc activation	$na1\_Bmal1$	4.13
Hill coeffificient for mBmal1 transcription due to REV_ERBα inhibition	$ni1\_Bmal1$	0.02
Hill coeffificient for mRorc transcription due to CLK_BMAL1 activation	$na1\_Rorc$	1.57
Hill coeffificient for mRorc transcription due to RORc activation	$na2\_Rorc$	0.56
Hill coeffificient for mRorc transcription due to PER1_CRY1 inhibition	$ni1\_Rorc$	2.25
Hill coeffificient for mRorc transcription due to PER1_CRY2 inhibition	$ni2\_Rorc$	4.73
Hill coeffificient for mRorc transcription due to PER2_CRY1 inhibition	$ni3\_Rorc$	3.43
Hill coeffificient for mRorc transcription due to PER2_CRY2 inhibition	$ni4\_Rorc$	0.84
Hill coeffificient for mRorc transcription due to REV_ERBα inhibition	$ni5\_Rorc$	4.33
Hill coeffificient for mRorc transcription due to REV_ERBα inhibition	$ni5\_Rorc$	4.33
Michaelis constant for mPer1 transcription due to CLK_BMAL1 activation	$KA1_{P1}$	1.98nM
Michaelis constant for mPer1 transcription due to PER1_CRY1 inhibition	$KI1_{P1}$	1.07nM
Michaelis constant for mPer1 transcription due to PER1_CRY2 inhibition	$KI2_{P1}$	3.96nM

Continued on next page

A3 Table continued

Parameter name	Parameter symbol	Value
Michaelis constant for mPer1 transcription due to PER2_CRY1 inhibition	$KI3_{P1}$	1.68nM
Michaelis constant for mPer1 transcription due to PER2_CRY2 inhibition	$KI4_{P1}$	3.11nM
Michaelis constant for mPer2 transcription due to CLK_BMAL1 activation	$KA1_{P2}$	1.90nM
Michaelis constant for mPer2 transcription due to PER1_CRY1 inhibition	$KI1_{P2}$	4.51nM
Michaelis constant for mPer2 transcription due to PER1_CRY2 inhibition	$KI2_{P2}$	2.98nM
Michaelis constant for mPer2 transcription due to PER2_CRY1 inhibition	$KI3_{P2}$	2.24nM
Michaelis constant for mPer2 transcription due to PER2_CRY2 inhibition	$KI4_{P2}$	3.31nM
Michaelis constant for mCry1 transcription due to CLK_BMAL1 activation	$KA1_{C1}$	1.46nM
Michaelis constant for mCry1 transcription due to RORc activation	$KA2_{C1}$	3.76nM
Michaelis constant for mCry1 transcription due to PER1_CRY1 inhibition	$KI1_{C1}$	0.03nM
Michaelis constant for mCry1 transcription due to PER1_CRY2 inhibition	$KI2_{C1}$	0.77nM
Michaelis constant for mCry1 transcription due to PER2_CRY1 inhibition	$KI3_{C1}$	3.59nM
Michaelis constant for mCry1 transcription due to PER2_CRY2 inhibition	$KI4_{C1}$	3.44nM
Michaelis constant for mCry1 transcription due to REV_ERBα inhibition	$KI5_{C1}$	2.82nM
Michaelis constant for mCry2 transcription due to CLK_BMAL1 activation	$KA1_{C2}$	0.69nM
Michaelis constant for mCry2 transcription due to RORc activation	$KA2_{C2}$	2.96nM
Michaelis constant for mCry2 transcription due to PER1_CRY1 inhibition	$KI1_{C2}$	4.63nM
Michaelis constant for mCry2 transcription due to PER1_CRY2 inhibition	$KI2_{C2}$	2.95nM

Continued on next page

A3 Table continued

Parameter name	Parameter symbol	Value
Michaelis constant for mCry2 transcription due to PER2_CRY1 inhibition	$KI3_{C2}$	3.57nM
Michaelis constant for mCry2 transcription due to PER2_CRY2 inhibition	$KI4_{C2}$	2.75nM
Michaelis constant for mCry2 transcription due to REV_ERBα inhibition	$KI5_{C2}$	3.97nM
Michaelis constant for mRev_erbα transcription due to CLK_BMAL1 activation	$KA1_{Re}$	3.15nM
Michaelis constant for mRev_erbα transcription due to PER1_CRY1 inhibition	$KI1_{Re}$	3.56nM
Michaelis constant for mRev_erbα transcription due to PER1_CRY2 inhibition	$KI2_{Re}$	3.62nM
Michaelis constant for mRev_erbα transcription due to PER2_CRY1 inhibition	$KI3_{Re}$	4.71nM
Michaelis constant for mRev_erbα transcription due to PER2_CRY2 inhibition	$KI4_{Re}$	1.23nM
Michaelis constant for mClk transcription due to RORc activation	$KA1_{Clk}$	1.59nM
Michaelis constant for mClk transcription due to REV_ERBα inhibition	$KI1_{Clk}$	0.83nM
Michaelis constant for mBmal1 transcription due to RORc activation	$KA1_{Bmal1}$	2.59nM
Michaelis constant for mBmal1 transcription due to REV_ERBα inhibition	$KI1_{Bmal1}$	2.47nM
Michaelis constant for mRorc transcription due to CLK_BMAL1 activation	$KA1_{Rorc}$	4.30nM
Michaelis constant for mRorc transcription due to RORc activation	$KA2_{Rorc}$	4.89nM
Michaelis constant for mRorc transcription due to PER1_CRY1 inhibition	$KI1_{Rorc}$	3.49nM
Michaelis constant for mRorc transcription due to PER1_CRY2 inhibition	$KI2_{Rorc}$	2.34nM
Michaelis constant for mRorc transcription due to PER2_CRY1 inhibition	$KI3_{Rorc}$	2.71nM
Michaelis constant for mRorc transcription due to PER2_CRY2 inhibition	$KI4_{Rorc}$	2.09nM

Continued on next page

A3 Table continued

Parameter name	Parameter symbol	Value
Michaelis constant for mRorc transcription due to REV_ERBα inhibition	$KI5_{Rorc}$	3.36nM
Degradation rate of mPer1 mRNA	km_{P1}	2.18/h
Degradation rate of mPer2 mRNA	km_{P2}	0.20/h
Degradation rate of mCry1 mRNA	km_{C1}	0.22/h
Degradation rate of mCry2 mRNA	km_{C2}	0.41/h
Degradation rate of mRev_erbα mRNA	km_{Re}	0.60/h
Degradation rate of mClk mRNA	km_{Clk}	3.19/h
Degradation rate of mBmal1 mRNA	km_{Bmal1}	1.42/h
Degradation rate of mRorc mRNA	km_{Rorc}	1.50/h
Translation rate for PER1 protein formation	t_P1	3.05/h
Translation rate for PER2 protein formation	t_P2	2.38/h
Translation rate for CRY1 protein formation	t_C1	3.94/h
Translation rate for CRY2 protein formation	t_C2	1.69/h
Translation rate for REV_ERBα protein formation	t_R_E	1.60/h
Translation rate for CLK protein formation	t_CLK	3.04/h
Translation rate for BMAL1 protein formation	t_Bmal1	4.00/h
Translation rate for RORc protein formation	t_Rorc	1.39/h
Degradation rate for PER1 protein	kp_P1	2.58/h
Degradation rate for PER2 protein	kp_P2	0.16/h
Degradation rate for CRY1 protein	kp_C1	3.03/h
Degradation rate for CRY2 protein	kp_C2	1.72/h
Degradation rate for REV_ERBα protein	kp_R_E	0.31/h
Degradation rate for CLK protein	kp_CLK	1.52/h
Degradation rate for BMAL1 protein	kp_BMAL1	2.28/h
Degradation rate for RORc protein	kp_RORc	3.33/h
Complex association rate for PER1_CRY1	a_P1C1	3.57/nM.hr
Complex association rate for PER1_CRY2	a_P1C2	3.12/nM.hr
Complex association rate for PER2_CRY1	a_P2C1	3.81/nM.hr
Complex association rate for PER2_CRY2	a_P2C2	0.95/nM.hr
Complex association rate for CLK_BMAL1	a_CLK_BMAL1	1.98/nM.hr
Complex dissociation rate for PER1_CRY1	d_P1C1	1.32/h
Complex dissociation rate for PER1_CRY2	d_P1C2	1.85/h
Complex dissociation rate for PER2_CRY1	d_P2C1	1.37/h
Complex dissociation rate for PER2_CRY2	d_P2C2	2.42/h

Continued on next page

A3 Table continued

Parameter name	Parameter symbol	Value
Complex dissociation rate for CLK_BMAL1	d_CLK_BMAL1	0.97/h
Complex association rate for HSF1_CLK_BMAL1	a_HBC	0.01/nM.hr
Complex dissociation rate for HSF1_CLK_BMAL1	d_HBC	0.01/nM.hr
Maximum rate of mPer2 transcription from HSF1 trimer activation	$V2_{HSF1\_3}$	0.4nM/h
Hill coeffificient for mPer2 transcription due to HSF1 trimer activation	$m3\_HSF1\_3$	30
Michaelis constant for mPer2 transcription due to HSF1 trimer activation	$K3_{HSF1\_3}$	0.03nM
The coefficient of temperature influence	k_{I}	60
Rate of Hsp substrate association	$k1$	25.2
Dissociation rate of Hsp substrate complexes	$l1$	0.3
Rate of Hsp HSF1 association	$k2$	25.2
Dissociation rate of Hsp HSF1 complexes	$l2$	0.3
Trimers formation rate (HSF1 activation rate)	$k3$	13.8
Trimers dissociation rate (HSF1 inactivation rate)	$l3$	4.83
Hsp translation rate	$k4$	2.1
Rate of HSF1 dissociation and formation of Hsp substrate	$k6$	1.38
Rate of substrate dissociation and formation of Hsp HSF1 complexes	$l6$	0.0216
Refolding rate	$k10$	0.84
Rate constant of Hsp degradation	$l10$	0.78
Translation rate	$k8$	2.1
Rate of active HSF1 HSE association	$k7$	29.4
Rate of active HSF1 HSE dissociation	$l7$	2.1

A4 Table Initial value for circadian clock model

Symbols	Initial value
mPer1	0.162262276671167
mPer2	0.132683679183005
mCry1	0.110749112097186
mCry2	0.941903733357527
mRev_erb α	0.0531686566299661
mClk	1.20981171325816
mBmal1	0.717979076502352
mRorc	0.0505093594221419
PER1	0.165886553016483
PER2	5.93687261056107
CRY1	0.344162992360903
CRY2	0.839815827093128
R_E	0.153009403719356
CLK	2.45247543928806
mBMAL1	1.27085829481866
RORc	0.0194298699438274
P1C1	0.148163123205506
P1C2	6.10954245265612
P2C1	0.214556454673887
P2C2	1.93325734526827
BC	6.39105280539227
HBC	0.139868933288505
HSF1	0.875483235275281
hsp	0.715119414419869
S	0.0534764439789149
hsp_HSF1	6.85519916116170
hsp_S	1.28571216610016
HSF1_3	0.0109477069992554
HSE	1.7345011343824
HSF1_3_HSE	0.265498865617223
mhsp	0.265514948080689

The peripheral oscillator produces sustained circadian expression profiles of the core clock genes (S1 Fig).

2 Equations, Parameters, and Initial Conditions for the Two-Compartment SCN Model

The SCN model consists of two individual single models: a Ventral SCN (c) and a Dorsal SCN (s). Unlike the single model, an AVP equation (eq(96)) is added to the Dorsal SCN, where AVP activates mPer1 and mPer2 in the Dorsal SCN. In the Ventral SCN, a VIP equation (eq(64)) is introduced, and VIP can activate mPer1 and mPer2 in both the Dorsal SCN and the Ventral SCN. The rest of the parameters and equations are the same as those in the single model. The specific equations are as follows(eq(33)–eq(96)):

$$\frac{dmPer1\_c}{dt} = V\_C \cdot \frac{VIP^{nac\_VIP}}{Kc_{VIP} + VIP^{nac\_VIP}} + \left(V0_{P1} + V1_{P1} \cdot \frac{BC\_c^{na1\_P1}}{KA1_{P1}^{na1\_P1} + BC\_c^{na1\_P1}}\right) \cdot \frac{KI1_{P1}^{ni1\_P1}}{KI1_{P1}^{ni1\_P1} + P1C1\_c^{ni1\_P1}} \cdot \frac{KI2_{P1}^{ni2\_P1}}{KI2_{P1}^{ni2\_P1} + P1C2\_c^{ni2\_P1}} \cdot \frac{KI3_{P1}^{ni3\_P1}}{KI3_{P1}^{ni3\_P1} + P2C1\_c^{ni3\_P1}} \cdot \frac{KI4_{P1}^{ni4\_P1}}{KI4_{P1}^{ni4\_P1} + P2C2\_c^{ni4\_P1}} - km_{P1} \cdot mPer1\_c \tag{33}$$

$$\frac{dmPer2\_c}{dt} = V\_C \cdot \frac{VIP^{nac\_VIP}}{Kc_{VIP} + VIP^{nac\_VIP}} + \left(V0_{P2} + V1_{P2} \cdot \frac{BC\_c^{na1\_P2}}{KA1_{P2}^{na1\_P2} + BC\_c^{na1\_P2}}\right) \cdot \frac{KI1_{P2}^{ni1\_P2}}{KI1_{P2}^{ni1\_P2} + P1C1\_c^{ni1\_P2}} \cdot \frac{KI2_{P2}^{ni2\_P2}}{KI2_{P2}^{ni2\_P2} + P1C2\_c^{ni2\_P2}} \cdot \frac{KI3_{P2}^{ni3\_P2}}{KI3_{P2}^{ni3\_P2} + P2C1\_c^{ni3\_P2}} \cdot \frac{KI4_{P2}^{ni4\_P2}}{KI4_{P2}^{ni4\_P2} + P2C2\_c^{ni4\_P2}} - km_{P2} \cdot mPer2\_c + \frac{V2_{hsf1\_3} \cdot HSF1\_3\_c^{m3\_hsf1\_3}}{K3_{hsf1\_3}^{m3\_hsf1\_3} + HSF1\_3\_c^{m3\_hsf1\_3}} \tag{34}$$

$$\frac{dmCry1\_c}{dt} = \left(V0_{C1} + \frac{V1_{C1} \cdot BC\_c^{na1\_C1}}{KA1_{C1}^{na1\_C1} + BC\_c^{na1\_C1}} + \frac{V2_{C1} \cdot RORc\_c^{na2\_C1}}{KA2_{C1}^{na2\_C1} + RORc\_c^{na2\_C1}}\right) \cdot \frac{KI1_{C1}^{ni1\_C1}}{KI1_{C1}^{ni1\_C1} + P1C1\_c^{ni1\_C1}} \cdot \frac{KI2_{C1}^{ni2\_C1}}{KI2_{C1}^{ni2\_C1} + P1C2\_c^{ni2\_C1}} \cdot \frac{KI3_{C1}^{ni3\_C1}}{KI3_{C1}^{ni3\_C1} + P2C1\_c^{ni3\_C1}} \cdot \frac{KI4_{C1}^{ni4\_C1}}{KI4_{C1}^{ni4\_C1} + P2C2\_c^{ni4\_C1}} \cdot \frac{KI5_{C1}^{ni5\_C1}}{KI5_{C1}^{ni5\_C1} + R\_E\_c^{ni5\_C1}} - km_{C1} \cdot mCry1\_c \tag{35}$$

$$\frac{dmCry2\_c}{dt} = \left(V0_{C2} + \frac{V1_{C2} \cdot BC\_c^{na1\_C2}}{KA1_{C2}^{na1\_C2} + BC\_c^{na1\_C2}} + \frac{V2_{C2} \cdot RORc\_c^{na2\_C2}}{KA2_{C2}^{na2\_C2} + RORc\_c^{na2\_C2}}\right) \cdot \frac{KI1_{C2}^{ni1\_C2}}{KI1_{C2}^{ni1\_C2} + P1C1\_c^{ni1\_C2}} \cdot \frac{KI2_{C2}^{ni2\_C2}}{KI2_{C2}^{ni2\_C2} + P1C2\_c^{ni2\_C2}} \cdot \frac{KI3_{C2}^{ni3\_C2}}{KI3_{C2}^{ni3\_C2} + P2C1\_c^{ni3\_C2}} \cdot \frac{KI4_{C2}^{ni4\_C2}}{KI4_{C2}^{ni4\_C2} + P2C2\_c^{ni4\_C2}} \cdot \frac{KI5_{C2}^{ni5\_C2}}{KI5_{C2}^{ni5\_C2} + R\_E\_c^{ni5\_C2}} - km_{C2} \cdot mCry2\_c \tag{36}$$

$$\frac{dmRev\_erb\alpha\_c}{dt} = \frac{V1_{Re} \cdot BC\_c^{na1\_Re}}{KA1_{Re}^{na\_Re} + BC\_c^{na1\_Re}} \cdot \frac{KI1_{Re}^{ni1\_Re}}{KI1_{Re}^{ni1\_Re} + P1C1\_c^{ni1\_Re}}$$
$$\cdot \frac{KI2_{Re}^{ni2\_Re}}{KI2_{Re}^{ni2\_Re} + P1C2\_c^{ni2\_Re}} \cdot \frac{KI3_{Re}^{ni3\_Re}}{KI3_{Re}^{ni3\_Re} + P2C1\_c^{ni3\_Re}}$$
$$\cdot \frac{KI4_{Re}^{ni4\_Re}}{KI4_{Re}^{ni4\_Re} + P2C2\_c^{ni4\_Re}} - km_{Re} \cdot mRev\_erb\alpha\_c \quad (37)$$

$$\frac{dmClk\_c}{dt} = \left(V0_{Clk} + \frac{V1_{Clk} \cdot RORc\_c^{na1\_Clk}}{KA1_{Clk}^{na1\_Clk} + RORc\_c^{na1\_Clk}}\right) \cdot \left(\frac{KI1_{Clk}^{ni1_{Clk}}}{KI1_{Clk}^{ni1_{Clk}} + R_E\_c^{ni1_{Clk}}}\right)$$
$$- km_{Clk} \cdot mClk\_c \quad (38)$$

$$\frac{dmBmal1\_c}{dt} = \left(V0_{Bmal1} + \frac{V1_{Bmal1} \cdot RORc\_c^{na1\_Bmal1}}{KA1_{Bmal1}^{na1\_Bmal1} + RORc\_c^{na1\_Bmal1}}\right)$$
$$\cdot \frac{KI1_{Bmal1}^{ni1\_Bmal1}}{KI1_{Bmal1}^{ni1\_Bmal1} + R\_E\_c^{ni1\_Bmal1}} - km_{Bmal1} \cdot mBmal1\_c \quad (39)$$

$$\frac{dmRorc\_c}{dt} = \left(V0_{Rorc} + \frac{V1_{Rorc} \cdot BC\_c^{na1\_Rorc}}{KA1_{Rorc}^{na1\_Rorc} + BC\_c^{na1\_Rorc}} + \frac{V2_{Rorc} \cdot RORc\_c^{na2\_Rorc}}{KA2_{Rorc}^{na2\_Rorc} + RORc\_c^{na2\_Rorc}}\right)$$
$$\cdot \frac{KI1_{Rorc}^{ni1\_Rorc}}{KI1_{Rorc}^{ni1\_Rorc} + P1C1\_c^{ni1\_Rorc}} \cdot \frac{KI2_{Rorc}^{ni2\_Rorc}}{KI2_{Rorc}^{ni2\_Rorc} + P1C2\_c^{ni2\_Rorc}}$$
$$\cdot \frac{KI3_{Rorc}^{ni3\_Rorc}}{KI3_{Rorc}^{ni3\_Rorc} + P2C1\_c^{ni3\_Rorc}} \cdot \frac{KI4_{Rorc}^{ni4\_Rorc}}{KI4_{Rorc}^{ni4\_Rorc} + P2C2\_c^{ni4\_Rorc}}$$
$$\cdot \frac{KI5_{Rorc}^{ni5\_Rorc}}{KI5_{Rorc}^{ni5\_Rorc} + R\_E\_c^{ni5\_Rorc}} - km_{Rorc} \cdot mRorc\_c \quad (40)$$

$$\frac{dPER1\_c}{dt} = t\_P1 \cdot mPer1\_c - a\_P1C1 \cdot PER1\_c \cdot CRY1\_c - a\_P1C2 \cdot PER1\_c$$
$$\cdot CRY2\_c + d\_P1C1 \cdot P1C1\_c + d\_P1C2 \cdot P1C2\_c - kp\_P1 \cdot PER1\_c \quad (41)$$

$$\frac{dPER2\_c}{dt} = t\_P2 \cdot mPer2\_c - a\_P2C1 \cdot PER2\_c \cdot CRY1\_c - a\_P2C2 \cdot PER2\_c$$
$$\cdot CRY2\_c + d\_P2C1 \cdot P2C1\_c + d\_P2C2 \cdot P2C2\_c - kp\_P2 \cdot PER2\_c \quad (42)$$

$$\frac{dCRY1\_c}{dt} = t\_C1 \cdot mCry1\_c - a\_P1C1 \cdot PER1\_c \cdot CRY1\_c - a\_P2C1 \cdot PER2\_c$$
$$\cdot CRY1\_c + d\_P1C1 \cdot P1C1\_c + d\_P2C1 \cdot P2C1\_c - kp\_C1 \cdot CRY1\_c \quad (43)$$

$$\frac{dCRY2\_c}{dt} = t\_C2 \cdot mCry2\_c - a\_P1C2 \cdot PER1\_c \cdot CRY2\_c - a\_P2C2 \cdot PER2\_c$$
$$\cdot CRY2\_c + d\_P1C2 \cdot P1C2\_c + d\_P2C2 \cdot P2C2\_c - kp\_C2 \cdot CRY2\_c \quad (44)$$

$$\frac{dR\_E\_c}{dt} = t_{REV\_ERB} \cdot mRev\_erb\alpha\_c - kp\_REV\_ERB \cdot R\_E\_c \quad (45)$$

$$\frac{dCLK\_c}{dt} = t\_CLK \cdot mClk\_c - a\_CLK\_BMAL1 \cdot CLK\_c \cdot BMAL1\_c$$
$$+ d\_CLK\_BMAL1 \cdot BC\_c - kp\_CLK \cdot CLK\_c \quad (46)$$

$$\frac{dBMAL1\_c}{dt} = t\_Bmal1 \cdot mBmal1\_c - a\_CLK\_BMAL1 \cdot CLK\_c \cdot BMAL1\_c$$
$$+ d\_CLK\_BMAL1 \cdot BC\_c - kp\_BMAL1 \cdot BMAL1\_c \quad (47)$$

$$\frac{dRORc\_c}{dt} = t\_Rorc \cdot mRorc\_c - kp\_RORc \cdot RORc\_c \tag{48}$$

$$\frac{dP1C1\_c}{dt} = a\_P1C1 \cdot PER1\_c \cdot CRY1\_c - d\_P1C1 \cdot P1C1\_c \tag{49}$$

$$\frac{dP2C1\_c}{dt} = a\_P2C1 \cdot PER2\_c \cdot CRY1\_c - d\_P2C1 \cdot P2C1\_c \tag{50}$$

$$\frac{dP1C2\_c}{dt} = a\_P1C2 \cdot PER1\_c \cdot CRY2\_c - d\_P1C2 \cdot P1C2\_c \tag{51}$$

$$\frac{dP2C2\_c}{dt} = a\_P2C2 \cdot PER2\_c \cdot CRY2\_c - d\_P2C2 \cdot P2C2\_c \tag{52}$$

$$\frac{dBC\_c}{dt} = a\_CLK\_BMAL1 \cdot CLK\_c \cdot BMAL1\_c - d\_CLK\_BMAL1 \cdot BC\_c$$
$$- a\_HSF1\_BMAL1\_CLK \cdot BC\_c \cdot HSF1\_c + d\_HSF1\_BMAL1\_CLK \cdot HBC\_c \tag{53}$$

$$\frac{dHBC\_c}{dt} = a\_HSF1\_BMAL1\_CLK \cdot BC\_c \cdot HSF1\_c - d\_HSF1\_BMAL1\_CLK$$
$$\cdot HBC\_c \tag{54}$$

$$\frac{dHSF1\_c}{dt} = -k1 \cdot hsp\_c \cdot HSF1\_c + l1 \cdot hsp\_HSF1\_c - 3 \cdot k3 \cdot HSF1\_c^3$$
$$+ 2 \cdot l3 \cdot hsp\_c \cdot HSF1\_3\_c + k6 \cdot hsp\_HSF1\_c \cdot S\_c - l6 \cdot hsp\_S\_c \cdot HSF1\_c$$
$$- a\_HSF1\_BMAL1\_CLK \cdot BC\_c \cdot HSF1\_c + d\_HSF1\_BMAL1\_CLK \cdot HBC\_c \tag{55}$$

$$\frac{dhsp\_c}{dt} = -k1 \cdot hsp\_c \cdot HSF1\_c + l1 \cdot hsp\_HSF1\_c - k2 \cdot hsp\_c \cdot S\_c + l2 \cdot hsp\_S\_c$$
$$- l3 \cdot hsp\_c \cdot HSF1\_3\_c + k4 \cdot mhsp\_c + k10 \cdot hsp\_S\_c - l10 \cdot hsp\_c \tag{56}$$

$$\frac{dS\_c}{dt} = l2 \cdot hsp\_S\_c - k2 \cdot hsp\_c \cdot S\_c - k6 \cdot hsp\_HSF1\_c \cdot S\_c + l6 \cdot hsp\_S\_c \cdot HSF1\_c$$
$$+ \mathrm{I} \tag{57}$$

$$\frac{dhsp\_HSF1\_c}{dt} = k1 \cdot hsp\_c \cdot HSF1\_c - l1 \cdot hsp\_HSF1\_c - k6 \cdot hsp\_HSF1\_c \cdot S\_c$$
$$+ l6 \cdot hsp\_S\_c \cdot HSF1\_c + l3 \cdot hsp\_c \cdot HSF1\_3\_c \tag{58}$$

$$\frac{dhsp\_S\_c}{dt} = k2 \cdot hsp\_c \cdot S\_c - l2 \cdot hsp\_S\_c + k6 \cdot hsp\_HSF1\_c \cdot S\_c$$
$$- l6 \cdot hsp\_S\_c \cdot HSF1\_c - k10 \cdot hsp\_S\_c \tag{59}$$

$$\frac{dHSF1\_3\_c}{dt} = k3 \cdot HSF1\_c^3 - l3 \cdot hsp\_c \cdot HSF1\_3\_c - k7 \cdot HSF1\_3\_c \cdot HSE\_c$$
$$+ l7 \cdot HSF1\_3\_HSE\_c \tag{60}$$

$$\frac{dHSE\_c}{dt} = -k7 \cdot HSF1\_3\_c \cdot HSE\_c + l7 \cdot HSF1\_3\_HSE\_c \tag{61}$$

$$\frac{dHSF1\_3\_HSE\_c}{dt} = k7 \cdot HSF1\_3\_c \cdot HSE\_c - l7 \cdot HSF1\_3\_HSE\_c \tag{62}$$

$$\frac{dmhsp\_c}{dt} = k8 \cdot HSF1\_3\_HSE\_c - k4 \cdot mhsp\_c \tag{63}$$

$$\frac{dVIP}{dt} = t\_VIP \cdot (P1C2\_c + P1C1\_c + P2C2\_c + P2C1\_c) - kd\_VIP \cdot VIP \tag{64}$$

$$\frac{dmPer1\_s}{dt} = V\_S \cdot \frac{VIP^{nas\_VIP}}{Ks_{VIP} + VIP^{nas\_VIP}} + A\_S \cdot \frac{AVP^{nas\_AVP}}{Ks_{AVP} + AVP^{nas\_AVP}}$$

$$+\left(V0_{P1}+V1_{P1}\cdot\frac{BC\_s^{na1\_P1}}{KA1_{P1}^{na1\_P1}+BC\_s^{na1\_P1}}\right)\cdot\frac{KI1_{P1}^{ni1\_P1}}{KI1_{P1}^{ni1\_P1}+P1C1\_s^{ni1\_P1}}\cdot$$

$$\frac{KI2_{P1}^{ni2\_P1}}{KI2_{P1}^{ni2\_P1}+P1C2\_s^{ni2\_P1}}\cdot\frac{KI3_{P1}^{ni3\_P1}}{KI3_{P1}^{ni3\_P1}+P2C1\_s^{ni3\_P1}}\cdot\frac{KI4_{P1}^{ni4\_P1}}{KI4_{P1}^{ni4\_P1}+P2C2\_s^{ni4\_P1}}$$

$$-km_{P1}\cdot mPer1\_s \tag{65}$$

$$\frac{dmPer2\_s}{dt}=V\_S\cdot\frac{VIP^{nas\_VIP}}{Ks_{VIP}+VIP^{nas\_VIP}}+A\_S\cdot\frac{AVP^{nas\_AVP}}{Ks_{AVP}+AVP^{nas\_AVP}}$$

$$+\left(V0_{P2}+V1_{P2}\cdot\frac{BC\_s^{na1\_P2}}{KA1_{P2}^{na1\_P2}+BC\_s^{na1\_P2}}\right)\cdot\frac{KI1_{P2}^{ni1\_P2}}{KI1_{P2}^{ni1\_P2}+P1C1\_s^{ni1\_P2}}\cdot$$

$$\frac{KI2_{P2}^{ni2\_P2}}{KI2_{P2}^{ni2\_P2}+P1C2\_s^{ni2\_P2}}\cdot\frac{KI3_{P2}^{ni3\_P2}}{KI3_{P2}^{ni3\_P2}+P2C1\_s^{ni3\_P2}}\cdot\frac{KI4_{P2}^{ni4\_P2}}{KI4_{P2}^{ni4\_P2}+P2C2\_s^{ni4\_P2}}$$

$$-km_{P2}\cdot mPer2\_s+\frac{V2_{hsf1\_3}\cdot HSF1\_3\_s^{m3\_hsf1\_3}}{K3_{hsf1\_3}^{m3\_hsf1\_3}+HSF1\_3\_s^{m3\_hsf1\_3}} \tag{66}$$

$$\frac{dmCry1\_s}{dt}=\left(V0_{C1}+\frac{V1_{C1}\cdot BC\_s^{na1\_C1}}{KA1_{C1}^{na1\_C1}+BC\_s^{na1\_C1}}+\frac{V2_{C1}\cdot RORc\_s^{na2\_C1}}{KA2_{C1}^{na2\_C1}+RORc\_s^{na2\_C1}}\right)$$

$$\cdot\frac{KI1_{C1}^{ni1\_C1}}{KI1_{C1}^{ni1\_C1}+P1C1\_s^{ni1\_C1}}\cdot\frac{KI2_{C1}^{ni2\_C1}}{KI2_{C1}^{ni2\_C1}+P1C2\_s^{ni2\_C1}}\cdot\frac{KI3_{C1}^{ni3\_C1}}{KI3_{C1}^{ni3\_C1}+P2C1\_s^{ni3\_C1}}$$

$$\cdot\frac{KI4_{C1}^{ni4\_C1}}{KI4_{C1}^{ni4\_C1}+P2C2\_s^{ni4\_C1}}\cdot\frac{KI5_{C1}^{ni5\_C1}}{KI5_{C1}^{ni5\_C1}+R\_E\_s^{ni5\_C1}}-km_{C1}\cdot mCry1\_s \tag{67}$$

$$\frac{dmCry2\_s}{dt}=\left(V0_{C2}+\frac{V1_{C2}\cdot BC\_s^{na1\_C2}}{KA1_{C2}^{na1\_C2}+BC\_s^{na1\_C2}}+\frac{V2_{C2}\cdot RORc\_s^{na2\_C2}}{KA2_{C2}^{na2\_C2}+RORc\_s^{na2\_C2}}\right)$$

$$\cdot\frac{KI1_{C2}^{ni1\_C2}}{KI1_{C2}^{ni1\_C2}+P1C1\_s^{ni1\_C2}}\cdot\frac{KI2_{C2}^{ni2\_C2}}{KI2_{C2}^{ni2\_C2}+P1C2\_s^{ni2\_C2}}\cdot\frac{KI3_{C2}^{ni3\_C2}}{KI3_{C2}^{ni3\_C2}+P2C1\_s^{ni3\_C2}}$$

$$\cdot\frac{KI4_{C2}^{ni4\_C2}}{KI4_{C2}^{ni4\_C2}+P2C2\_s^{ni4\_C2}}\cdot\frac{KI5_{C2}^{ni5\_C2}}{KI5_{C2}^{ni5\_C2}+R\_E\_s^{ni5\_C2}}-km_{C2}\cdot mCry2\_s \tag{68}$$

$$\frac{dmRev\_erb\alpha\_s}{dt}=\frac{V1_{Re}\cdot BC\_s^{na1\_Re}}{KA1_{Re}^{na\_Re}+BC\_s^{na1\_Re}}\cdot\frac{KI1_{Re}^{ni1\_Re}}{KI1_{Re}^{ni1\_Re}+P1C1\_s^{ni1\_Re}}$$

$$\cdot\frac{KI2_{Re}^{ni2\_Re}}{KI2_{Re}^{ni2\_Re}+P1C2\_s^{ni2\_Re}}\cdot\frac{KI3_{Re}^{ni3\_Re}}{KI3_{Re}^{ni3\_Re}+P2C1\_s^{ni3\_Re}}$$

$$\cdot\frac{KI4_{Re}^{ni4\_Re}}{KI4_{Re}^{ni4\_Re}+P2C2\_s^{ni4\_Re}}-km_{Re}\cdot mRev\_erb\alpha\_s \tag{69}$$

$$\frac{dmClk\_s}{dt}=\left(V0_{Clk}+\frac{V1_{Clk}\cdot RORc\_s^{na1\_Clk}}{KA1_{Clk}^{na1\_Clk}+RORc\_s^{na1\_Clk}}\right)\cdot\left(\frac{KI1_{Clk}^{ni1_{Clk}}}{KI1_{Clk}^{ni1_{Clk}}+R_{E}\_s^{ni1_{Clk}}}\right)$$

$$-km_{Clk}\cdot mClk\_s \tag{70}$$

$$\frac{dmBmal1\_s}{dt}=\left(V0_{Bmal1}+\frac{V1_{Bmal1}\cdot RORc\_s^{na1\_Bmal1}}{KA1_{Bmal1}^{na1\_Bmal1}+RORc\_s^{na1\_Bmal1}}\right)$$

$$\cdot\frac{KI1_{Bmal1}^{ni1\_Bmal1}}{KI1_{Bmal1}^{ni1\_Bmal1}+R\_E\_s^{ni1\_Bmal1}}-km_{Bmal1}\cdot mBmal1\_s \tag{71}$$

$$\frac{dmRorc\_s}{dt} = \left(V0_{Rorc} + \frac{V1_{Rorc} \cdot BC\_s^{na1\_Rorc}}{KA1_{Rorc}^{na1\_Rorc} + BC\_s^{na1\_Rorc}} + \frac{V2_{Rorc} \cdot RORc\_s^{na2\_Rorc}}{KA2_{Rorc}^{na2\_Rorc} + RORc\_s^{na2\_Rorc}}\right)$$
$$\cdot \frac{KI1_{Rorc}^{ni1\_Rorc}}{KI1_{Rorc}^{ni1\_Rorc} + P1C1\_s^{ni1\_Rorc}} \cdot \frac{KI2_{Rorc}^{ni2\_Rorc}}{KI2_{Rorc}^{ni2\_Rorc} + P1C2\_s^{ni2\_Rorc}}$$
$$\cdot \frac{KI3_{Rorc}^{ni3\_Rorc}}{KI3_{Rorc}^{ni3\_Rorc} + P2C1\_s^{ni3\_Rorc}} \cdot \frac{KI4_{Rorc}^{ni4\_Rorc}}{KI4_{Rorc}^{ni4\_Rorc} + P2C2\_s^{ni4\_Rorc}}$$
$$\cdot \frac{KI5_{Rorc}^{ni5\_Rorc}}{KI5_{Rorc}^{ni5\_Rorc} + R\_E\_s^{ni5\_Rorc}} - km_{Rorc} \cdot mRorc\_s \tag{72}$$

$$\frac{dPER1\_s}{dt} = t\_P1 \cdot mPer1\_s - a\_P1C1 \cdot PER1\_s \cdot CRY1\_s - a\_P1C2 \cdot PER1\_s$$
$$\cdot CRY2\_s + d\_P1C1 \cdot P1C1\_s + d\_P1C2 \cdot P1C2\_s - kp\_P1 \cdot PER1\_s \tag{73}$$

$$\frac{dPER2\_s}{dt} = t\_P2 \cdot mPer2\_s - a\_P2C1 \cdot PER2\_s \cdot CRY1\_s - a\_P2C2 \cdot PER2\_s$$
$$\cdot CRY2\_s + d\_P2C1 \cdot P2C1\_s + d\_P2C2 \cdot P2C2\_s - kp\_P2 \cdot PER2\_s \tag{74}$$

$$\frac{dCRY1\_s}{dt} = t\_C1 \cdot mCry1\_s - a\_P1C1 \cdot PER1\_s \cdot CRY1\_s - a\_P2C1 \cdot PER2\_s$$
$$\cdot CRY1\_s + d\_P1C1 \cdot P1C1\_s + d\_P2C1 \cdot P2C1\_s - kp\_C1 \cdot CRY1\_s \tag{75}$$

$$\frac{dCRY2\_s}{dt} = t\_C2 \cdot mCry2\_s - a\_P1C2 \cdot PER1\_s \cdot CRY2\_s - a\_P2C2 \cdot PER2\_s$$
$$\cdot CRY2\_s + d\_P1C2 \cdot P1C2\_s + d\_P2C2 \cdot P2C2\_s - kp\_C2 \cdot CRY2\_s \tag{76}$$

$$\frac{dR\_E\_s}{dt} = t_{REV\_ERB} \cdot mRev\_erb\alpha\_s - kp\_REV\_ERB \cdot R\_E\_s \tag{77}$$

$$\frac{dCLK\_s}{dt} = t\_CLK \cdot mClk\_s - a\_CLK\_BMAL1 \cdot CLK\_s \cdot BMAL1\_s$$
$$+ d\_CLK\_BMAL1 \cdot BC\_s - kp\_CLK \cdot CLK\_s \tag{78}$$

$$\frac{dBMAL1\_s}{dt} = t\_Bmal1 \cdot mBmal1\_s - a\_CLK\_BMAL1 \cdot CLK\_s \cdot BMAL1\_s$$
$$+ d\_CLK\_BMAL1 \cdot BC\_s - kp\_BMAL1 \cdot BMAL1\_s \tag{79}$$

$$\frac{dRORc\_s}{dt} = t\_Rorc \cdot mRorc\_s - kp\_RORc \cdot RORc\_s \tag{80}$$

$$\frac{dP1C1\_s}{dt} = a\_P1C1 \cdot PER1\_s \cdot CRY1\_s - d\_P1C1 \cdot P1C1\_s \tag{81}$$

$$\frac{dP2C1\_s}{dt} = a\_P2C1 \cdot PER2\_s \cdot CRY1\_s - d\_P2C1 \cdot P2C1\_s \tag{82}$$

$$\frac{dP1C2\_s}{dt} = a\_P1C2 \cdot PER1\_s \cdot CRY2\_s - d\_P1C2 \cdot P1C2\_s \tag{83}$$

$$\frac{dP2C2\_s}{dt} = a\_P2C2 \cdot PER2\_s \cdot CRY2\_s - d\_P2C2 \cdot P2C2\_s \tag{84}$$

$$\frac{dBC\_s}{dt} = a\_CLK\_BMAL1 \cdot CLK\_s \cdot BMAL1\_s - d\_CLK\_BMAL1 \cdot BC\_s$$
$$- a\_HSF1\_BMAL1\_CLK \cdot BC\_s \cdot HSF1\_s + d\_HSF1\_BMAL1\_CLK \cdot HBC\_s \tag{85}$$

$$\frac{dHBC\_s}{dt} = a\_HSF1\_BMAL1\_CLK \cdot BC\_s \cdot HSF1\_s - d\_HSF1\_BMAL1\_CLK \cdot HBC\_s \quad (86)$$

$$\frac{dHSF1\_s}{dt} = -k1 \cdot hsp\_s \cdot HSF1\_s + l1 \cdot hsp\_HSF1\_s - 3 \cdot k3 \cdot HSF1\_s^3 + 2 \cdot l3 \cdot hsp\_s \cdot HSF1\_3\_s + k6 \cdot hsp\_HSF1\_s \cdot S\_s - l6 \cdot hsp\_S\_s \cdot HSF1\_s - a\_HSF1\_BMAL1\_CLK \cdot BC\_s \cdot HSF1\_s + d\_HSF1\_BMAL1\_CLK \cdot HBC\_s \quad (87)$$

$$\frac{dhsp\_s}{dt} = -k1 \cdot hsp\_s \cdot HSF1\_s + l1 \cdot hsp\_HSF1\_s - k2 \cdot hsp\_s \cdot S\_s + l2 \cdot hsp\_S\_s - l3 \cdot hsp\_s \cdot HSF1\_3\_s + k4 \cdot mhsp\_s + k10 \cdot hsp\_S\_s - l10 \cdot hsp\_s \quad (88)$$

$$\frac{dS\_s}{dt} = l2 \cdot hsp\_S\_s - k2 \cdot hsp\_s \cdot S\_s - k6 \cdot hsp\_HSF1\_s \cdot S\_s + l6 \cdot hsp\_S\_s \cdot HSF1\_s + \mathrm{I} \quad (89)$$

$$\frac{dhsp\_HSF1\_s}{dt} = k1 \cdot hsp\_s \cdot HSF1\_s - l1 \cdot hsp\_HSF1\_s - k6 \cdot hsp\_HSF1\_s \cdot S\_s + l6 \cdot hsp\_S\_s \cdot HSF1\_s + l3 \cdot hsp\_s \cdot HSF1\_3\_s \quad (90)$$

$$\frac{dhsp\_S\_s}{dt} = k2 \cdot hsp\_s \cdot S\_s - l2 \cdot hsp\_S\_s + k6 \cdot hsp\_HSF1\_s \cdot S\_s - l6 \cdot hsp\_S\_s \cdot HSF1\_s - k10 \cdot hsp\_S\_s \quad (91)$$

$$\frac{dHSF1\_3\_s}{dt} = k3 \cdot HSF1\_s^3 - l3 \cdot hsp\_s \cdot HSF1\_3\_s - k7 \cdot HSF1\_3\_s \cdot HSE\_s + l7 \cdot HSF1\_3\_HSE\_s \quad (92)$$

$$\frac{dHSE\_s}{dt} = -k7 \cdot HSF1\_3\_s \cdot HSE\_s + l7 \cdot HSF1\_3\_HSE\_s \quad (93)$$

$$\frac{dHSF1\_3\_HSE\_s}{dt} = k7 \cdot HSF1\_3\_s \cdot HSE\_s - l7 \cdot HSF1\_3\_HSE\_s \quad (94)$$

$$\frac{dmhsp\_s}{dt} = k8 \cdot HSF1\_3\_HSE\_s - k4 \cdot mhsp\_s \quad (95)$$

$$\frac{dAVP}{dt} = t\_AVP \cdot (P1C2\_s + P1C1\_s + P2C2\_s + P2C1\_s) - kd\_AVP \cdot AVP \quad (96)$$

A5 Table The definitions and values for the SCN model

Parameter name	Parameter symbol	Value
Maximum rate of mPer1 and mPer2 transcription in ventral SCN from VIP activation	V_C	0.002nM/h
Maximum rate of mPer1 and mPer2 transcription in dorsal SCN from VIP activation	V_S	0.001nM/h
Maximum rate of mPer1 and mPer2 transcription in dorsal SCN from AVP activation	A_S	0.001nM/h

Continued on next page

A5 Table continued

Parameter name	Parameter symbol	Value
The rate of VIP secretion	t_VIP	0.1/h
The rate of AVP secretion	t_AVP	0.1/h
Degradation rate for VIP	kd_VIP	1.5/h
Degradation rate for AVP	kd_AVP	1.5/h
Hill coeffificient for mPer1 and mPer2 transcription in ventral SCN due to VIP activation	nac_VIP	3
Hill coeffificient for mPer1 and mPer2 transcription in dorsal SCN due to VIP activation	nas_VIP	2
Hill coeffificient for mPer1 and mPer2 transcription in dorsal SCN due to AVP activation	nas_AVP	2
Michaelis constant for mPer1 and mPer2 transcription in ventral SCN due to VIP activation	Kc_VIP	1nM
Michaelis constant for mPer1 and mPer2 transcription in dorsal SCN due to VIP activation	Ks_VIP	1nM
Michaelis constant for mPer1 and mPer2 transcription in dorsal SCN due to AVP activation	Ks_AVP	1nM

A6 Table Initial value for the SCN model

Symbols	Initial value
mPer1_c	0.0141800294903420
mPer2_c	0.369382296340715
mCry1_c	0.549096314274231
mCry2_c	1.28459227599132
mRev_erb_c α	0.00227461814502857
mClk_c	1.24489979273252
mBmal1_c	0.725942939339851
mRorc_c	0.0249490570471889
PER1_c	0.0232221547771347
PER2_c	5.96156201505761
CRY1_c	0.488231058375081
CRY2_c	1.85849424279354
R_E_c	0.0340962231698350
CLK_c	2.46474330907698
mBMAL1_c	1.25732805185551
RORc_c	0.0119390441862457
P1C1_c	0.0303069219484425
P1C2_c	7.61728774019806
P2C1_c	0.0822347006602400
P2C2_c	4.64483334274294
BC_c	6.29139790382736
HBC_c	0.141841246081469
HSF1_c	0.887349525787102
hsp_c	0.729175392665905
S_c	0.0521018594481170
hsp_HSF1_c	7.12472058504629
hsp_S_c	1.28571576332609
HSF1_3_c	0.0111875639314793
HSE_c	1.72915801623674
HSF1_3_HSE_c	0.270841983763273
mhsp_c	0.270850977898567
VIP	0.821269023514921
mPer1_s	0.160977248875392
mPer2_s	0.135694370970586

Continued on next page

A6 Table continued

Symbols	Initial value
mCry1_s	0.112326343689597
mCry2_s	0.937042592955021
mRev_erb_s α	0.0513962270686008
mClk_s	1.21280543984038
mBmal1_s	0.718289949182856
mRorc_s	0.0496768319572133
PER1_s	0.164636597786280
PER2_s	5.96524662549162
CRY1_s	0.344527266944377
CRY2_s	0.840367113035979
R_E_s	0.146691006477117
CLK_s	2.45591856783305
mBMAL1_s	1.27060586087385
RORc_s	0.0191239914275129
P1C1_s	0.147014002797479
P1C2_s	6.13926839311831
P2C1_s	0.213369018840329
P2C2_s	1.94615482551800
BC_s	6.39650606305329
HBC_s	0.141829133159630
HSF1_s	0.888832340989551
hsp_s	0.729094826943930
S_s	0.0521081176622031
hsp_HSF1_s	7.12368149358801
hsp_S_s	1.28571235616849
HSF1_3_s	0.0111861924886275
HSE_s	1.72930051506767
HSF1_3_HSE_s	0.270699484932280
mhsp_s	0.270707077965617
AVP	0.586106251660226

The global SCN exhibits sustained circadian mRNA expression profiles of the core clock genes (S2 Fig).

B Robustness Analysis of Parameters Using Latin Hypercube Sampling

To evaluate the robustness of the model to parameter variations, 21 parameters controlling VIP/AVP signaling, Hill regulation, and formation of the $\mathrm{H\_B\_C}$ complex were independently varied using Latin hypercube sampling (LHS). For each perturbation level, the marginal range of each parameter was divided into 10,000 equal-probability strata, one value was sampled from each stratum, and the strata were independently permuted across parameters. For parameter p_i, the sampling range was defined as

$$p_i \in \left[(1-r)p_i^0, (1+r)p_i^0\right], \qquad r = 0.10 \text{ or } 0.20, \tag{97}$$

where p_i^0 denotes the nominal parameter value. Each analysis therefore contained 10,000 Latin hypercube samples together with the nominal parameter set, giving a total of 10,001 parameter sets. B1 Table summarizes the parameters and their corresponding nominal values and perturbation ranges.

B1 Table Parameters used in the 21-dimensional sensitivity analysis.

Parameter	Meaning	Nominal	10% range	20% range
t_{VIP}	VIP production rate	0.1	0.09–0.11	0.08–0.12
kd_{VIP}	VIP degradation rate	1.5	1.35–1.65	1.2–1.8
V_C	VIP input strength to ventral SCN	0.002	0.0018–0.0022	0.0016–0.0024
$K_{\mathrm{VIP},c,P1}$	VIP Hill constant for ventral SCN *Per1*	1	0.9–1.1	0.8–1.2
$n_{\mathrm{VIP},c,P1}$	VIP Hill coefficient for ventral SCN *Per1*	3	2.7–3.3	2.4–3.6
$K_{\mathrm{VIP},c,P2}$	VIP Hill constant for ventral SCN *Per2*	1	0.9–1.1	0.8–1.2
$n_{\mathrm{VIP},c,P2}$	VIP Hill coefficient for ventral SCN *Per2*	3	2.7–3.3	2.4–3.6
V_S	VIP input strength to dorsal SCN	0.001	0.0009–0.0011	0.0008–0.0012
$K_{\mathrm{VIP},s,P1}$	VIP Hill constant for dorsal SCN *Per1*	1	0.9–1.1	0.8–1.2

Continued on next page

B1 Table continued

Parameter	Meaning	Nominal	10% range	20% range
$n_{\mathrm{VIP},s,P1}$	VIP Hill coefficient for dorsal SCN *Per1*	2	1.8–2.2	1.6–2.4
$K_{\mathrm{VIP},s,P2}$	VIP Hill constant for dorsal SCN *Per2*	1	0.9–1.1	0.8–1.2
$n_{\mathrm{VIP},s,P2}$	VIP Hill coefficient for dorsal SCN *Per2*	2	1.8–2.2	1.6–2.4
t_{AVP}	AVP production rate	0.1	0.09–0.11	0.08–0.12
kd_{AVP}	AVP degradation rate	1.5	1.35–1.65	1.2–1.8
A_C	AVP input strength to dorsal SCN	0.001	0.0009–0.0011	0.0008–0.0012
$K_{\mathrm{AVP},s,P1}$	AVP Hill constant for dorsal SCN *Per1*	1	0.9–1.1	0.8–1.2
$n_{\mathrm{AVP},s,P1}$	AVP Hill coefficient for dorsal SCN *Per1*	2	1.8–2.2	1.6–2.4
$K_{\mathrm{AVP},s,P2}$	AVP Hill constant for dorsal SCN *Per2*	1	0.9–1.1	0.8–1.2
$n_{\mathrm{AVP},s,P2}$	AVP Hill coefficient for dorsal SCN *Per2*	2	1.8–2.2	1.6–2.4
$a_{\mathrm{H\_B\_C}}$	H_B_C association rate	0.01	0.009–0.011	0.008–0.012
$d_{\mathrm{H\_B\_C}}$	H_B_C dissociation rate	0.01	0.009–0.011	0.008–0.012

Each sampled parameter set was first integrated without stimulation for 1,200 h and was subsequently evaluated for stable synchronization in non-overlapping 240-h windows for up to 10,000 additional hours. Peaks were detected directly from the regional output signals. For each region $q \in \{\mathrm{VL}, \mathrm{DM}\}$, the adjacent-peak period and cycle amplitude were calculated as

$$P_{q,k} = t^{\mathrm{peak}}_{q,k+1} - t^{\mathrm{peak}}_{q,k}, \qquad A_{q,k} = \max_{[t^{\mathrm{peak}}_{q,k},\, t^{\mathrm{peak}}_{q,k+1}]} \mathit{Per2}_q - \min_{[t^{\mathrm{peak}}_{q,k},\, t^{\mathrm{peak}}_{q,k+1}]} \mathit{Per2}_q. \tag{98}$$

A parameter set was classified as synchronized only when, in the final stable 240-h window, both regions simultaneously satisfied

$$\begin{gathered} \mathrm{range}(P_q) < 0.05\ \mathrm{h}, \quad \mathrm{range}(A_q) < 0.05, \quad q \in \{\mathrm{VL}, \mathrm{DM}\}, \\ \left|\overline{P}_{\mathrm{VL}} - \overline{P}_{\mathrm{DM}}\right| < 10^{-9}\ \mathrm{h}. \end{gathered} \tag{99}$$

Here, $\mathrm{range}(Z) = \max_k Z_k - \min_k Z_k$, and $\overline{P}_q$ denotes the mean period in that window. To quantify the relative phase relationship between the two SCN regions, the first ventral SCN peak following each dorsal SCN peak was identified. With P denoting the median

regional period, the signed dorsal-to-ventral SCN phase difference was calculated as

$$\phi_{\mathrm{DM}\to\mathrm{VL}} = \mathrm{wrap}_P\left(t^{\mathrm{peak}}_{\mathrm{VL,next}} - t^{\mathrm{peak}}_{\mathrm{DM}}\right), \qquad \phi_{\mathrm{DM}\to\mathrm{VL}} \in [-P/2, P/2). \tag{100}$$

Here, $\mathrm{wrap}_P(z) = z - P\lfloor (z + P/2)/P \rfloor$ maps a time difference into $[-P/2, P/2)$. Positive values indicate that the dorsal SCN leads the ventral SCN, whereas negative values indicate that the ventral SCN leads the dorsal SCN.

Using these criteria, 6,649 of the 10,001 parameter sets were retained in the 10% analysis, including 6,458 dorsal-leading and 191 ventral-leading sets. In the 20% analysis, 3,523 sets were retained, including 3,201 dorsal-leading and 322 ventral-leading sets. Most phase differences clustered at a dorsal SCN lead of 8.5–9 h, whereas a smaller population showed signed ventral-leading differences from -11.8 to -6 h (S4 Fig).

Phase-response curves were subsequently calculated for the synchronized parameter sets using ventral SCN *Per2*, dorsal SCN *Per2*, and a weighted global SCN reporter defined as

$$S_{\mathrm{SCN}}(t) = \frac{1}{3}Per2_{\mathrm{VL}}(t) + \frac{2}{3}Per2_{\mathrm{DM}}(t). \tag{101}$$

Before stimulation, the unstimulated global SCN peak was aligned to CT9, and a 2-h heat pulse at $39.1\,^{\circ}\mathrm{C}$ was applied at each integer CT from CT0 to CT23. The global SCN phase was quantified from the timing of troughs in the global SCN signal. Trough searching began 12 h after the end of stimulation. For the control trajectory, the lowest global SCN trough within the search interval was selected as the reference and classified according to the regional slope configuration: either the dorsal signal was rising while the ventral signal was falling, or vice versa. The stimulated trajectory could contain global SCN troughs with different regional configurations. The trough used for phase quantification was therefore not selected solely by its timing or depth. Instead, the first trough matching the regional configuration of the control reference trough was selected. After the corresponding control and stimulated global SCN troughs had been identified, the dorsal and ventral troughs nearest to each global trough were used to calculate the regional phase shifts. In a small number of cases, stimulation strongly distorted the global SCN waveform, and no trough with the control configuration occurred within the search interval. In such cases, a large trough-based global phase shift may reflect both displacement of the reference feature and reorganization of the composite waveform, and should therefore be interpreted cautiously. Such extreme responses may be less frequent in the intact SCN, where additional stabilizing mechanisms, including intercellular coupling, can constrain excessive regional phase shifts. These cases were excluded from the box summaries and their frequency was quantified separately in S5C Fig; for each signal $x \in \{\mathrm{VL}, \mathrm{DM}, \mathrm{SCN}\}$, the phase shift was calculated as

$$\Delta\phi_x = \mathrm{wrap}_P\left(t^{\mathrm{control}}_{x,\mathrm{trough}} - t^{\mathrm{pulse}}_{x,\mathrm{trough}}\right), \tag{102}$$

where positive values indicate phase advances and negative values indicate phase delays relative to the control trajectory.

Across both perturbation ranges, the global SCN phase shift generally lay between the ventral and dorsal shifts (S5A and S5B Fig; S6A and S6B Fig). The 70 synchronized sets with a 0.1–4 h regional phase difference exhibited larger global shifts than most other synchronized sets but retained this relationship at every CT (S7 Fig). S5C Fig gives the dorsal- and ventral-leading fractions, S5D Fig shows a representative CT22 case, and S6C Fig shows the corresponding 10% results.

C A Conceptual Two-Oscillator Model Based on Poincaré Oscillators

The SCN was represented by two coupled Poincaré (Stuart–Landau) oscillators, referred to here as the ventral-like and dorsal-like oscillators. As a reduced phenomenological model, it was not intended to capture the detailed molecular kinetics of either SCN subdivision; instead, we focused primarily on stimulus-induced changes in oscillator phase.

The ventral-like oscillator drives the dorsal-like oscillator unidirectionally. With $r_V^2 = x_V^2 + y_V^2$ and $r_D^2 = x_D^2 + y_D^2$, the model is

$$\dot{x}_V = \lambda x_V - \omega y_V - r_V^2 x_V + u_V(t), \tag{103}$$

$$\dot{y}_V = \omega x_V + \lambda y_V - r_V^2 y_V, \tag{104}$$

$$\dot{x}_D = \lambda x_D - \omega y_D - r_D^2 x_D + K\left(x_V^\delta - x_D\right) + u_D(t), \tag{105}$$

$$\dot{y}_D = \omega x_D + \lambda y_D - r_D^2 y_D + K\left(y_V^\delta - y_D\right), \tag{106}$$

where the subscripts V and D denote the ventral-like and dorsal-like oscillators, respectively. The phase-shifted ventral-like state entering the coupling term is

$$\begin{pmatrix} x_V^\delta \\ y_V^\delta \end{pmatrix} = \begin{pmatrix} \cos\delta & -\sin\delta \\ \sin\delta & \cos\delta \end{pmatrix} \begin{pmatrix} x_V \\ y_V \end{pmatrix}. \tag{107}$$

Thus, the phase difference is introduced by rotating the ventral-like state before it drives the dorsal-like oscillator. With $\delta = 2\pi/3$ and $\omega = 2\pi/24\ \mathrm{h}^{-1}$, the imposed phase offset is

$$\frac{\delta}{\omega} = 8\ \mathrm{h},$$

so that the dorsal-like oscillator leads the ventral-like oscillator by approximately 8 h in the unstimulated synchronized state.

A rectangular pulse beginning at circadian time t_s was applied additively to the x components. The simulated conditions were

$$(u_V, u_D) = \begin{cases} (A\chi_{[t_s,t_s+\tau]}(t), A\chi_{[t_s,t_s+\tau]}(t)), & \text{simultaneous stimulation of both oscillators,} \\ (A\chi_{[t_s,t_s+\tau]}(t), 0), & \text{ventral-like-only stimulation,} \\ (0,0), & \text{no-pulse control.} \end{cases} \tag{108}$$

Although the pulse enters only the Cartesian x equation, it can alter both amplitude and phase. Writing $x_j = r_j \cos\theta_j$ and $y_j = r_j \sin\theta_j$, the direct contributions of the stimulus are

$$\dot{r}_j\big|_{\text{pulse}} = u_j(t)\cos\theta_j, \qquad \dot{\theta}_j\Big|_{\text{pulse}} = -\frac{u_j(t)}{r_j}\sin\theta_j, \qquad j \in \{V, D\}. \tag{109}$$

The response therefore depends on the circadian phase at which the pulse is applied.

The oscillator-specific and composite signals used for analysis were

$$X_{\text{VL}} = x_V, \qquad X_{\text{DM}} = x_D, \qquad X_{\text{SCN}} = x_V + x_D. \tag{110}$$

Here, X_{VL} and X_{DM} denote the ventral-like and dorsal-like signals. The SCN-like signal is the direct sum of these two signals rather than the output of an additional oscillator.

The initial state $(1, 0, \cos\delta, \sin\delta)^{\text{T}}$ was relaxed for 200 h without stimulation. The relaxed trajectory was then rephased so that the maximum of the control X_{SCN} signal occurred at CT9. This anchoring defined CT0 and the circadian times used for stimulation.

C1 Table Model, stimulation, and numerical parameters.

Parameter	Value	Interpretation
λ	1.0	Linear radial growth rate
ω	$2\pi/24$ h^{-1}	Intrinsic angular frequency (24-h period)
δ	$2\pi/3$	Ventral-like-to-dorsal-like phase rotation (equivalent to 8 h)
K	0.01	Unidirectional ventral-like-to-dorsal-like coupling strength
A	0.3	Pulse amplitude applied to the x component
τ	1.5 h	Pulse duration

For each stimulus phase, control and stimulated trajectories were initialized from the same CT9-anchored state. Phase shifts were quantified using the same trough-based procedure described in Section 2, with trough searching beginning 12 h after stimulus onset.

The phase shift of signal x was calculated as

$$\Delta\phi_x = \text{wrap}_{24}\left(t_{x,\text{trough}}^{\text{control}} - t_{x,\text{trough}}^{\text{stimulated}}\right), \qquad x \in \{\text{VL}, \text{DM}, \text{SCN}\}, \tag{111}$$

where wrap_{24} maps the time difference to one circadian cycle, represented within $[-12, 12)$ h. Under this convention, positive values denote phase advances and negative values denote phase delays relative to the no-pulse control.

Across all stimulus phases, the phase shift of the composite SCN-like signal lay between the corresponding ventral-like and dorsal-like phase shifts (S8 Fig). This result

illustrates how combining two phase-offset oscillatory signals can moderate the phase response of their composite output.

For comparison with the detailed molecular SCN model, S9 Fig shows phase-response curves under simultaneous and ventral-only stimulation, whereas S10 Fig shows the corresponding acute *Per2* responses.
