# Supplementary material for: A compartmentalized SCN model explains temperature resistance through interregional phase differences
Source: PLoS Comput Biol. 2026 Sep 29;22(9):e1014831. doi: 10.1371/journal.pcbi.1014831 (PMC13645230; doi:10.1371/journal.pcbi.1014831)
Supplement: Supplementary file 2 [file pcbi.1014831.s002.pdf]

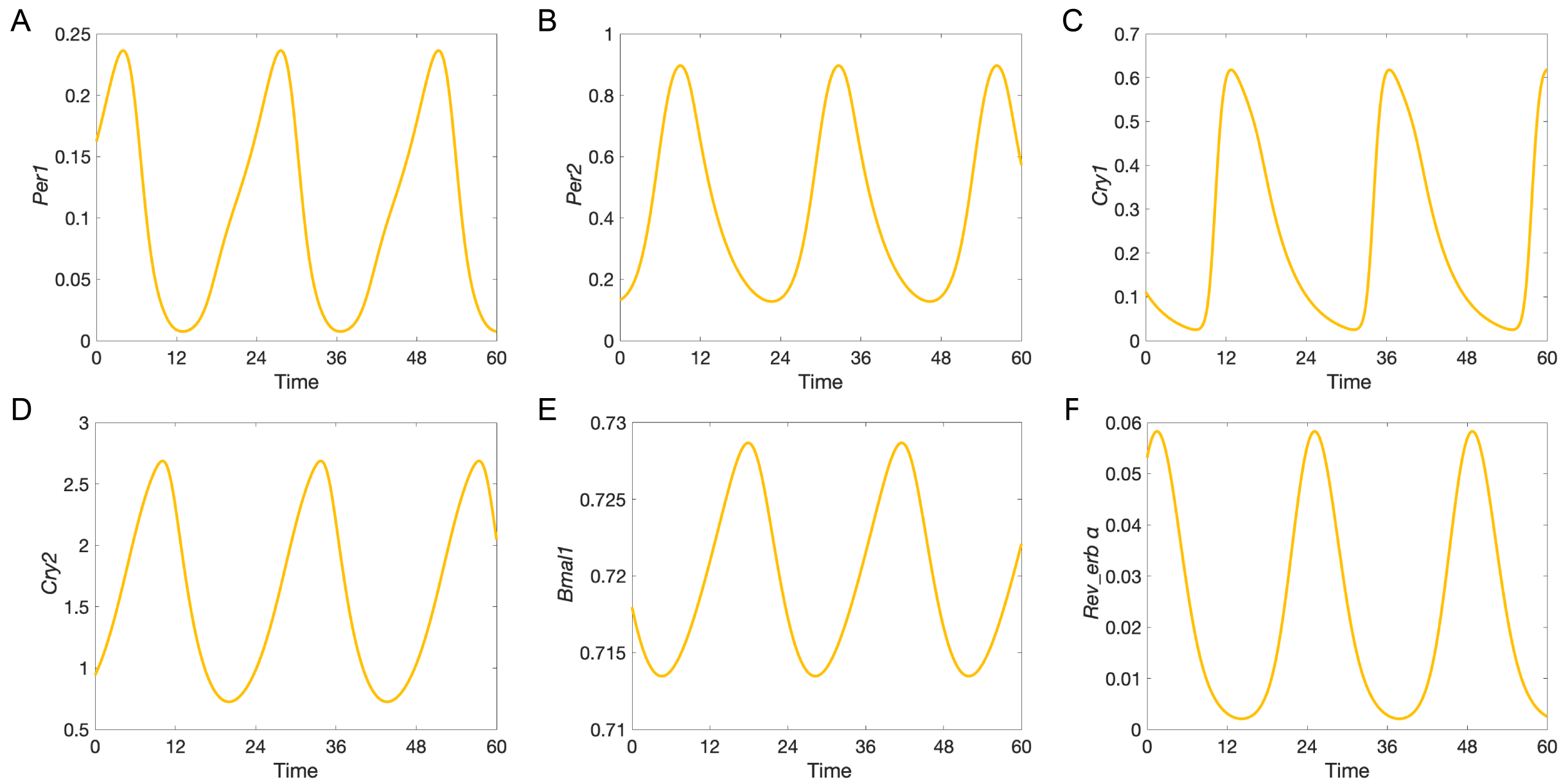

S1 Fig mRNA expression profiles of circadian genes in the peripheral oscillator model: (A) *Per1*, (B) *Per2*, (C) *Cry1*, (D) *Cry2*, (E) *Bmal1*, and (F) *Rev-erb*α.
