# Supplementary material for: A compartmentalized SCN model explains temperature resistance through interregional phase differences
Source: PLoS Comput Biol. 2026 Sep 29;22(9):e1014831. doi: 10.1371/journal.pcbi.1014831 (PMC13645230; doi:10.1371/journal.pcbi.1014831)
Supplement: Supplementary file 4 [file pcbi.1014831.s004.pdf]

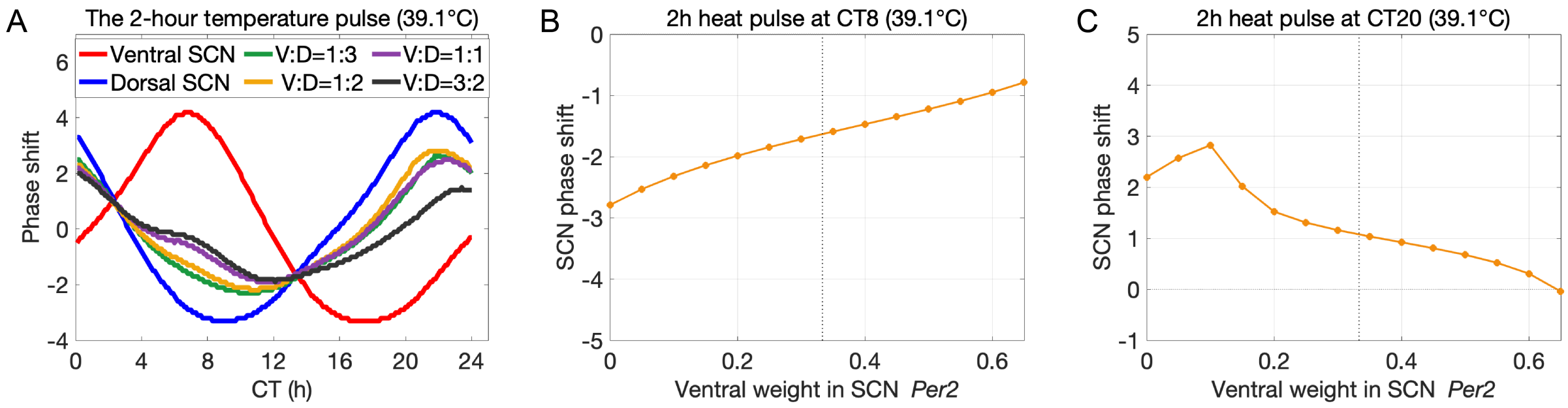

S3 Fig Effect of ventral–dorsal weighting on the global SCN phase response to a 2-h temperature pulse (39.1°C). (A) Phase-response curves for ventral:dorsal weighting ratios of 1:3 (emerald green), 1:2 (yellow), 1:1 (purple), and 3:2 (dark gray), together with the ventral (red) and dorsal (blue) SCN responses. (B,C) Global SCN phase shifts at CT20 and CT8, respectively, as a function of the ventral contribution to SCN *Per2* expression. Positive values denote phase advances and negative values denote phase delays relative to the no-pulse control.
