# Supplementary material for: A compartmentalized SCN model explains temperature resistance through interregional phase differences
Source: PLoS Comput Biol. 2026 Sep 29;22(9):e1014831. doi: 10.1371/journal.pcbi.1014831 (PMC13645230; doi:10.1371/journal.pcbi.1014831)
Supplement: Supplementary file 5 [file pcbi.1014831.s005.pdf]

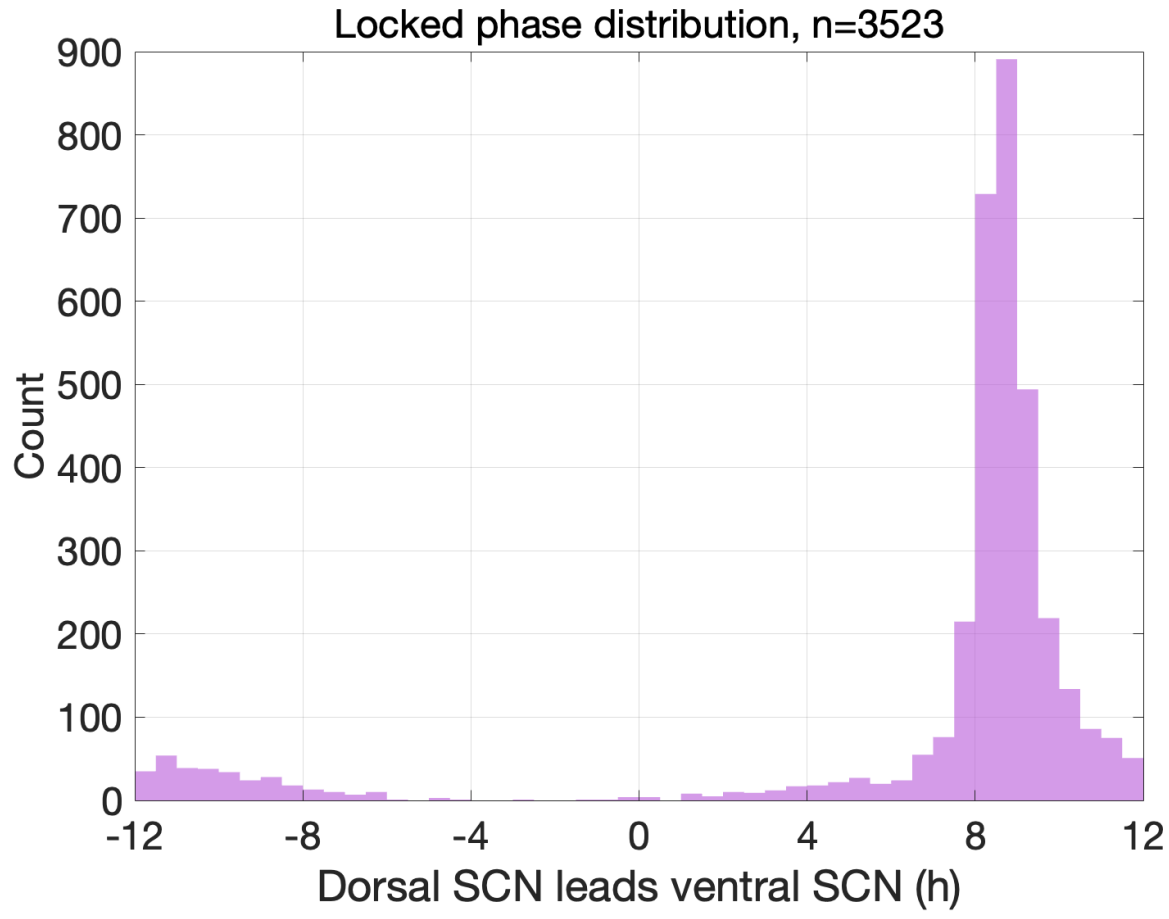

S4 Fig Distribution of the signed dorsal-to-ventral SCN phase difference among the synchronized parameter sets in the 20% analysis ($n = 3523$). Most sets cluster at a dorsal SCN lead of 8.5–9 h. Positive values indicate that the dorsal SCN leads the ventral SCN, whereas negative values indicate that the ventral SCN leads the dorsal SCN
