# Supplementary material for: A compartmentalized SCN model explains temperature resistance through interregional phase differences
Source: PLoS Comput Biol. 2026 Sep 29;22(9):e1014831. doi: 10.1371/journal.pcbi.1014831 (PMC13645230; doi:10.1371/journal.pcbi.1014831)
Supplement: Supplementary file 6 [file pcbi.1014831.s006.pdf]

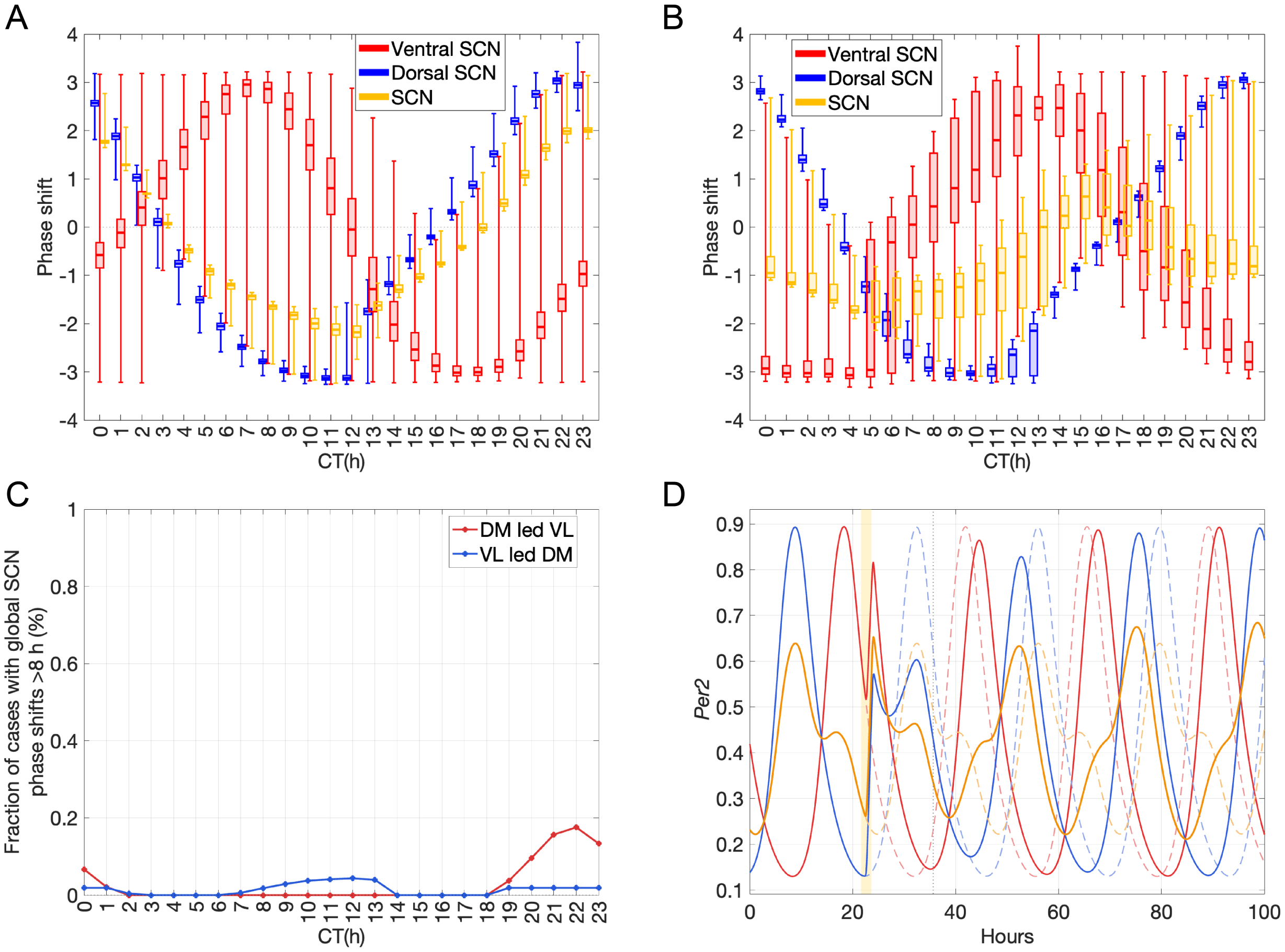

S5 Fig Global and subregional phase responses under ±20% perturbations of the newly introduced parameters. (A) PRCs for synchronized sets in which the dorsal SCN led the ventral SCN ($n = 3201$). (B) PRCs for sets in which the ventral SCN led the dorsal SCN ($n = 322$). In both groups, global SCN phase shifts generally lay between the corresponding ventral and dorsal shifts. Red, blue, and yellow denote ventral SCN, dorsal SCN, and weighted global SCN signals, respectively. Boxes show the interquartile range, center lines show the median, and whiskers span the finite values. (C) Fraction of synchronized cases with trough phase shifts exceeding 8 h at each CT, shown separately for dorsal-leading (red) and ventral-leading (blue) configurations. (D) Representative CT22 case from the >8-h category. In this example, the dorsal SCN undergoes a phase shift exceeding 20 h, such that it shifts from leading to lagging relative to the ventral SCN, producing a large change in the trough of the global SCN signal. In intact biological systems, such extreme shifts may be moderated by intercellular coupling. The yellow band marks the 2-h pulse. Dashed curves show the control condition, and solid curves show the pulse response; red, blue, and yellow denote the ventral SCN, dorsal SCN, and global SCN, respectively.
