# Supplementary material for: A compartmentalized SCN model explains temperature resistance through interregional phase differences
Source: PLoS Comput Biol. 2026 Sep 29;22(9):e1014831. doi: 10.1371/journal.pcbi.1014831 (PMC13645230; doi:10.1371/journal.pcbi.1014831)
Supplement: Supplementary file 7 [file pcbi.1014831.s007.pdf]

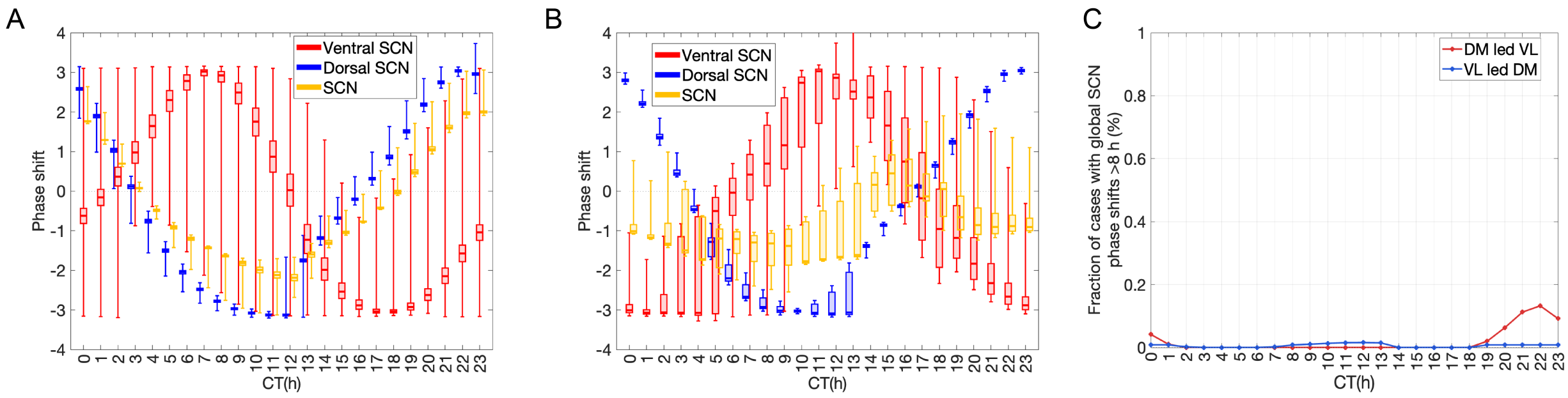

S6 Fig Global and subregional phase responses under $\pm 10\%$ perturbations of the newly introduced parameters. (A) PRCs for synchronized sets in which the dorsal SCN led the ventral SCN ($n = 6458$). (B) PRCs for sets in which the ventral SCN led the dorsal SCN ($n = 191$). Box definitions and colors are as in S5 Fig (C) Fraction of synchronized cases with trough phase shifts exceeding 8 h at each CT, shown separately for dorsal-leading (red) and ventral-leading (blue) configurations.
