# Supplementary material for: A compartmentalized SCN model explains temperature resistance through interregional phase differences
Source: PLoS Comput Biol. 2026 Sep 29;22(9):e1014831. doi: 10.1371/journal.pcbi.1014831 (PMC13645230; doi:10.1371/journal.pcbi.1014831)
Supplement: Supplementary file 8 [file pcbi.1014831.s008.pdf]

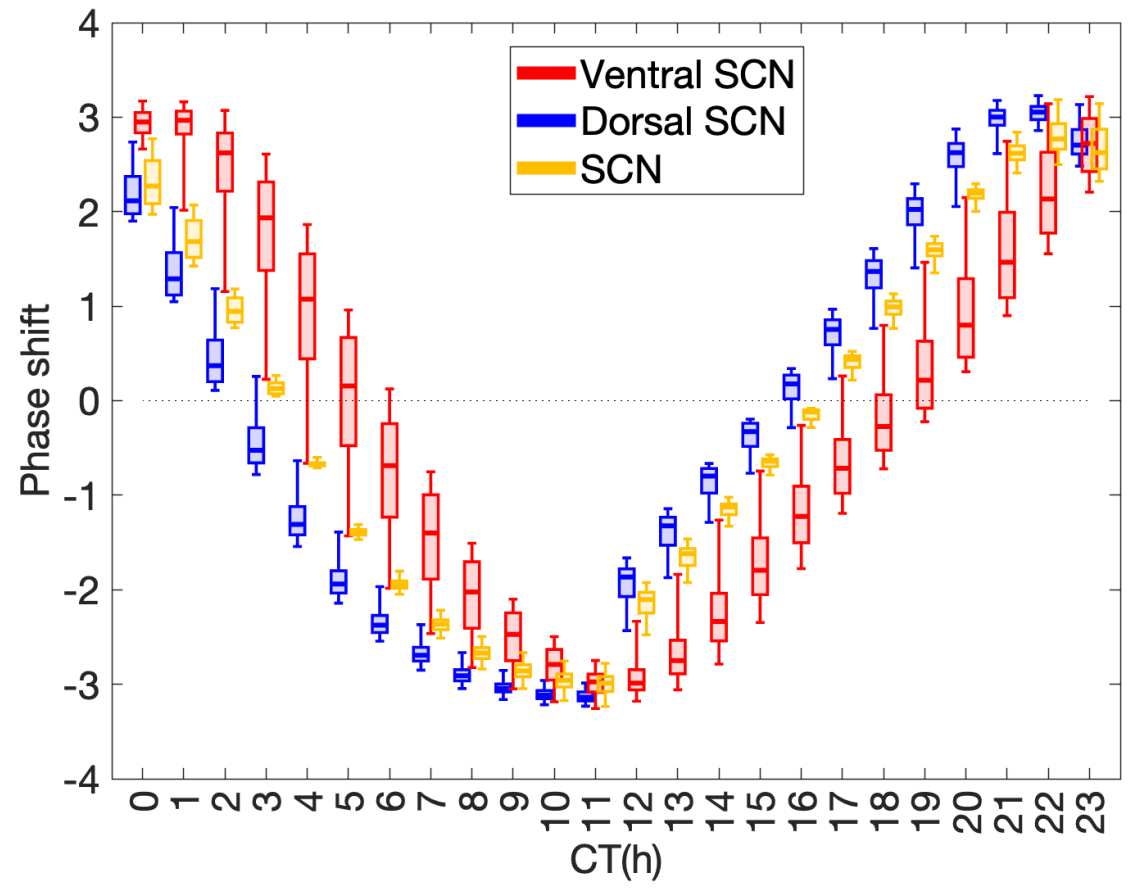

S7 Fig Subregional and global SCN phase-shift distributions across CT for synchronized parameter sets with small dorsal–ventral phase differences (0.1–4 h). All 70 parameter sets had a valid result at every CT. Red, blue, and yellow denote ventral SCN, dorsal SCN, and weighted global SCN signals. Boxes show the interquartile range, center lines show the median, whiskers span the finite values, and the horizontal dotted line denotes zero phase shift. At every CT, the global SCN phase shift lay between the corresponding dorsal and ventral SCN phase shifts.
