# Supplementary material for: A compartmentalized SCN model explains temperature resistance through interregional phase differences
Source: PLoS Comput Biol. 2026 Sep 29;22(9):e1014831. doi: 10.1371/journal.pcbi.1014831 (PMC13645230; doi:10.1371/journal.pcbi.1014831)
Supplement: Supplementary file 9 [file pcbi.1014831.s009.pdf]

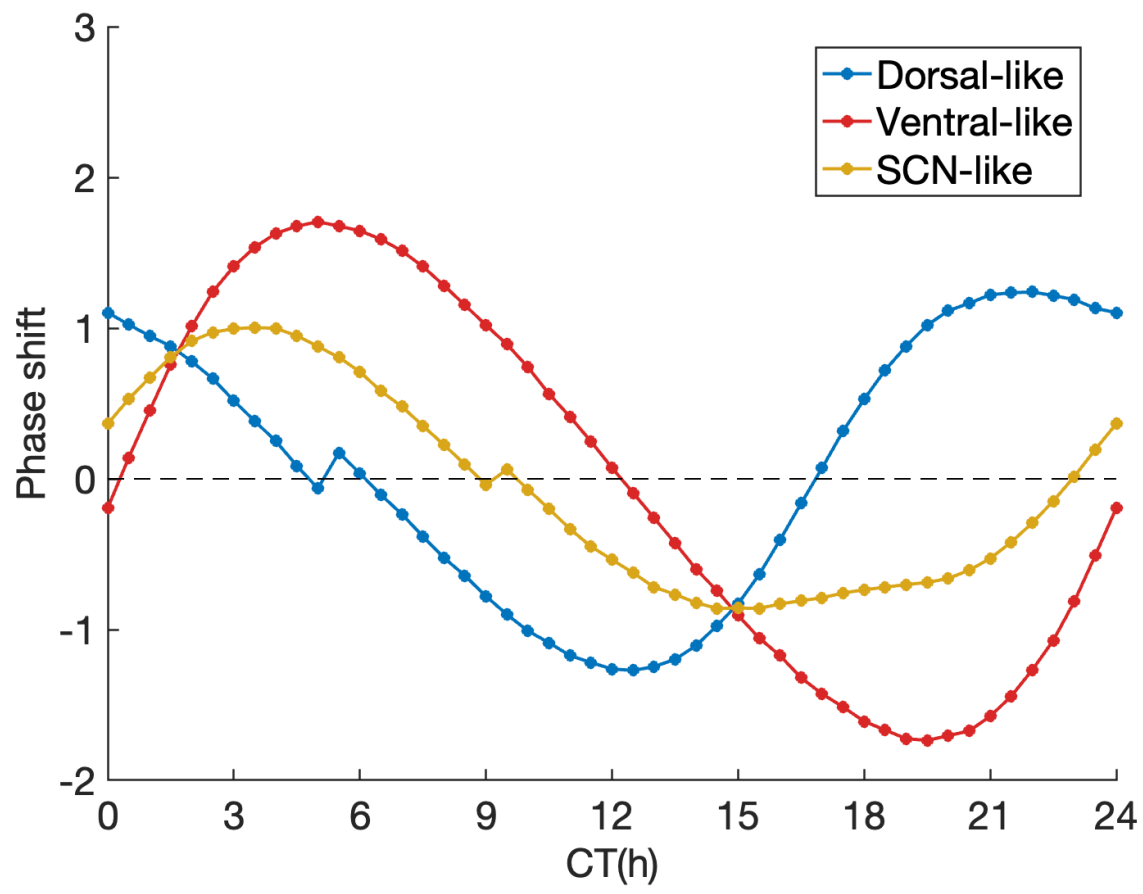

S8 Fig Phase-response curves in the two-oscillator Poincaré model under simultaneous stimulation of the dorsal-like and ventral-like oscillators. Blue, red, and yellow curves denote the dorsal-like, ventral-like, and global SCN-like signals, respectively. The 48 stimulus phases span CT0–CT23.5 in 0.5-h increments. The horizontal dashed line indicates zero phase shift. Positive values denote trough advances relative to the no-pulse control, and negative values denote delays.
