# Supplementary material for: A compartmentalized SCN model explains temperature resistance through interregional phase differences
Source: PLoS Comput Biol. 2026 Sep 29;22(9):e1014831. doi: 10.1371/journal.pcbi.1014831 (PMC13645230; doi:10.1371/journal.pcbi.1014831)
Supplement: Supplementary file 10 [file pcbi.1014831.s010.pdf]

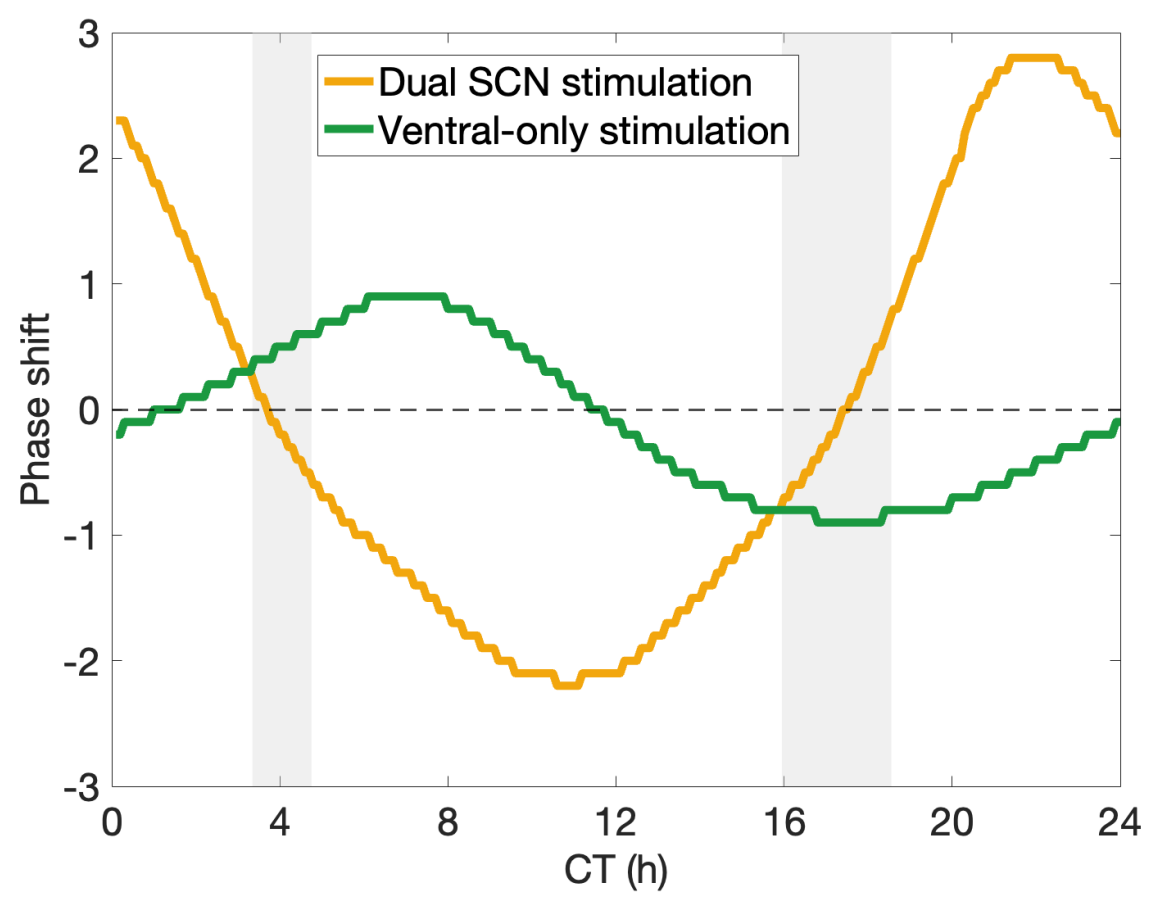

S9 Fig Phase-response curves of the compartmentalized SCN model under different spatial patterns of thermal stimulation. Phase shifts of the global SCN signal were calculated following a 2-hour heat pulse at 39.1°C applied at different circadian times. The yellow curve represents simultaneous stimulation of both the dorsal and ventral SCN (dual SCN stimulation), whereas the green curve represents stimulation of the ventral SCN alone (ventral-only stimulation). Gray shading indicates circadian phases at which dual stimulation produces a smaller absolute phase shift of the global SCN than ventral-only stimulation. These intervals lie within a phase range in which the dorsal and ventral SCN regions exhibit oppositely directed phase responses under dual stimulation, allowing partial cancellation of their contributions to the global SCN phase shift.
