# Supplementary material for: A compartmentalized SCN model explains temperature resistance through interregional phase differences
Source: PLoS Comput Biol. 2026 Sep 29;22(9):e1014831. doi: 10.1371/journal.pcbi.1014831 (PMC13645230; doi:10.1371/journal.pcbi.1014831)
Supplement: Supplementary file 11 [file pcbi.1014831.s011.pdf]

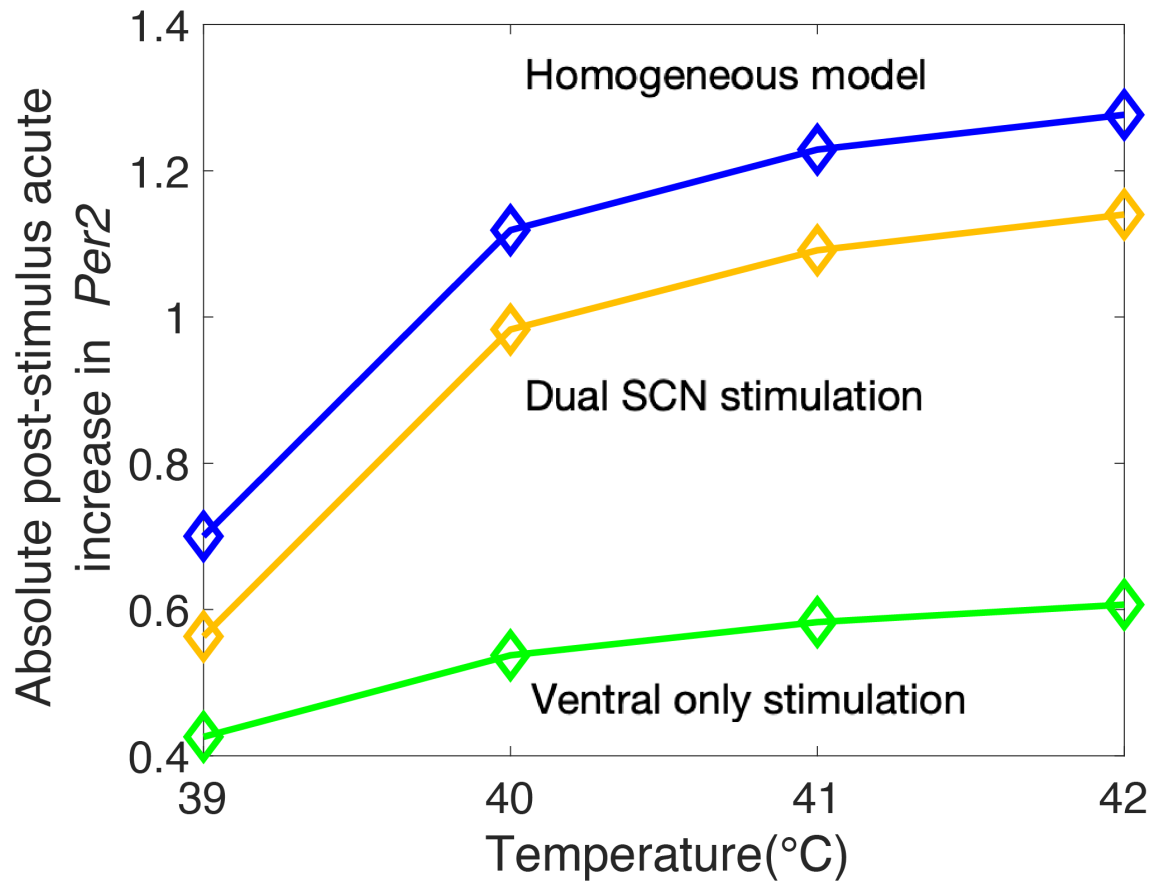

S10 Fig Difference in *Per2* expression after stimulation at different temperatures (39–42°C) at CT4 under three conditions: dual SCN stimulation (yellow), ventral-only stimulation (green), and the homogeneous model (blue). The difference was defined as the maximum *Per2* level after stimulation minus its value immediately before stimulation. Across the tested temperatures, dual stimulation consistently produced a substantially larger absolute increase in *Per2* than ventral-only stimulation, yet the global phase shift remained markedly smaller.
